# Nationwide annual agricultural land-use maps of Germany from 1990 to 2023 derived from satellite imagery

**DOI:** 10.1038/s41597-026-08375-w

**Published:** 2026-09-26

**Authors:** Gideon Okpoti Tetteh, Vu-Dong Pham, Marcel Schwieder, Lukas Blickensdörfer, Alexander Gocht, Sebastian van der Linden, Stefan Erasmi

**Affiliations:** 1 Thünen Institute of Farm Economics, Bundesallee 63, 38116 Braunschweig, Germany; 2 Earth Observation and Geoinformation Science Lab, Institute of Geography and Geology, University of Greifswald, Friedrich-Ludwig-Jahn-Str. 16, 17489 Greifswald, Germany; 3 Interdisciplinary Centre for Baltic Sea Region Research (IFZO), University of Greifswald, 17489 Greifswald, Germany; 4 Geography Department, Humboldt University of Berlin, Unter den Linden 6, 10099 Berlin, Germany; 5 Agricultural Development and Trade Group, Department of Agricultural Economics, Humboldt University of Berlin, Unter den Linden 6, 10099 Berlin, Germany

## Abstract

We present German-wide annual agricultural land-use maps from 1990 to 2023, created from Landsat and Sentinel-2 images using a deep learning approach. Based on farmers’ declarations from 2006 to 2022, we extracted annual training samples for 13 crop classes and one grassland class. These samples were used to train a multi-year one-dimensional convolutional neural network, which was subsequently applied to generate the annual land-use maps. Between 2010 and 2022, overall map accuracies ranged between 85% and 93%. When averaged over time, dominant classes like grassland, rapeseed, winter cereals, sugar beet, and maize were detected with high accuracy (≥90%). Conversely, minor classes such as fallow land and plantation were predicted with low accuracy (≤52%). Comparison of map areas with agricultural statistics over time revealed high correlations for most classes, particularly maize (*r* = 0.978). The presented maps provide an essential basis for analyzing long-term trends in agricultural land-use. They can be used to fill temporal gaps in national agricultural statistics and to disaggregate those statistics to higher spatial units.

## Background & Summary

In Germany, the land-use (LU) in agricultural landscapes has undergone significant changes over the past decades. The drivers behind those changes are multifaceted, reflecting a complex interplay of socio-economic, policy, and environmental factors^1–5^. These drivers have collectively shaped the agricultural landscape, influencing the extent, type, and intensity of agricultural LU. To design effective policies that foster sustainable agriculture, we need a comprehensive understanding of those drivers and their impact on agricultural LU. This requires long-term, spatially explicit information on agricultural LU.

Primary data sources such as the Authoritative Topographic-Cartographic Information System (ATKIS)^6^, the Geo-spatial Application (GSA) of the Common Agricultural Policy (CAP)^7^ framework, and official agricultural statistics^8^ are available in Germany for monitoring agricultural lands. However, those data sources have certain limitations. In ATKIS, only broad land-cover (LC) classes, such as cropland and grassland, are mapped, without detailed information on agricultural LU types. While detailed information on agricultural LU types can be found in the GSA, the data has limited spatial and temporal coverage, and it is not publicly available for all federal states. Although the official agricultural statistics contain detailed information on agricultural LU and productivity, a comprehensive farm survey is conducted only every 3-4 years at sample farm locations. Additionally, the survey data are aggregated to the district level, thereby preventing spatially explicit analysis of agricultural LU changes. Further, the LU definitions used in the statistics change over time, thereby requiring considerable effort to consolidate the LU classes.

Remote sensing data, with their high spatial and temporal coverage, are suitable for continuous and long-term area-wide mapping of agricultural lands^9,10^, thereby providing a unique means of overcoming the limitations of the abovementioned data sources. The literature is replete with the use of remote sensing data for generating agricultural LU maps at local, national, and continental scales^11,12^. The proliferation of agricultural LU maps has been driven by key developments, including the increasing volume of data from various satellite missions, advances in artificial intelligence methods for agricultural LU mapping, and greater access to all-in-one cloud computing platforms^10,12,13^.

The launch of Sentinel-1 in 2014 and Sentinel-2 in 2015, as part of the Copernicus satellite program, has accelerated the creation of national-scale agricultural LU maps across Europe and beyond. This is particularly true for Germany, where numerous nationwide products exist^14–20^. However, detailed national-scale agricultural LU maps for years before the Copernicus era, dating back to 1990, are rarely available, making effective long-term monitoring difficult. The year 1990 is pivotal because it marks the baseline year for mandatory national greenhouse gas (GHG) inventory reporting by countries, including Germany, that are parties to the United Nations Framework Convention on Climate Change (UNFCCC)^21^. Therefore, high-resolution, area-wide, and long-term LU maps in Germany will not only aid in understanding the drivers behind agricultural LU changes but may also improve national GHG emissions reporting, because the maps could potentially be used for Tier-3 (process-based) modelling^22,23^ as defined in the Intergovernmental Panel on Climate Change (IPCC) guidelines. Other potential uses of such long-term maps include the spatial and temporal disaggregation of agricultural statistics^24,25^, assessing agricultural landscape heterogeneity within the context of biodiversity monitoring^26,27^, analyzing crop sequences and rotations^28,29^, evaluating agricultural policies^30,31^, and estimating crop supply response^32^.

This study presents a historical dataset of agricultural LU maps for Germany, containing 13 main crops and one grassland class from 1990 to 2023, generated from the entire Landsat (1990–2023) and Sentinel-2 (2015–2023) archives using a deep learning (DL) approach. The combination of Landsat and Sentinel-2 from 2015 onwards yields a temporally dense time series^33,34^, which enables the differentiation of agricultural LU types based on their phenological profiles^35,36^. However, the time series before 2015 consists only of Landsat data and thus has lower spatial resolution and larger temporal gaps, making detailed mapping of agricultural LU more challenging. Further, LU mapping relies on reference data, which is needed for model training and validation. In more recent years, reference data such as the agricultural parcels declared by farmers in the GSA of CAP have become available in the European Union (EU)^37,38^. However, due to the limited spatial and temporal availability of the GSA data in Germany, long-term agricultural LU mapping has been hampered. For model training and validation in this study, we had access to GSA data covering different parts of Germany from 2006 to 2022. To overcome the lack of reference data before 2006 and the temporarily sparse satellite data before 2015, we adapted the DL approach of Pham et al.^39^, which was designed for LU mapping in data-sparse conditions.

## Methods

**Fig. 1:**
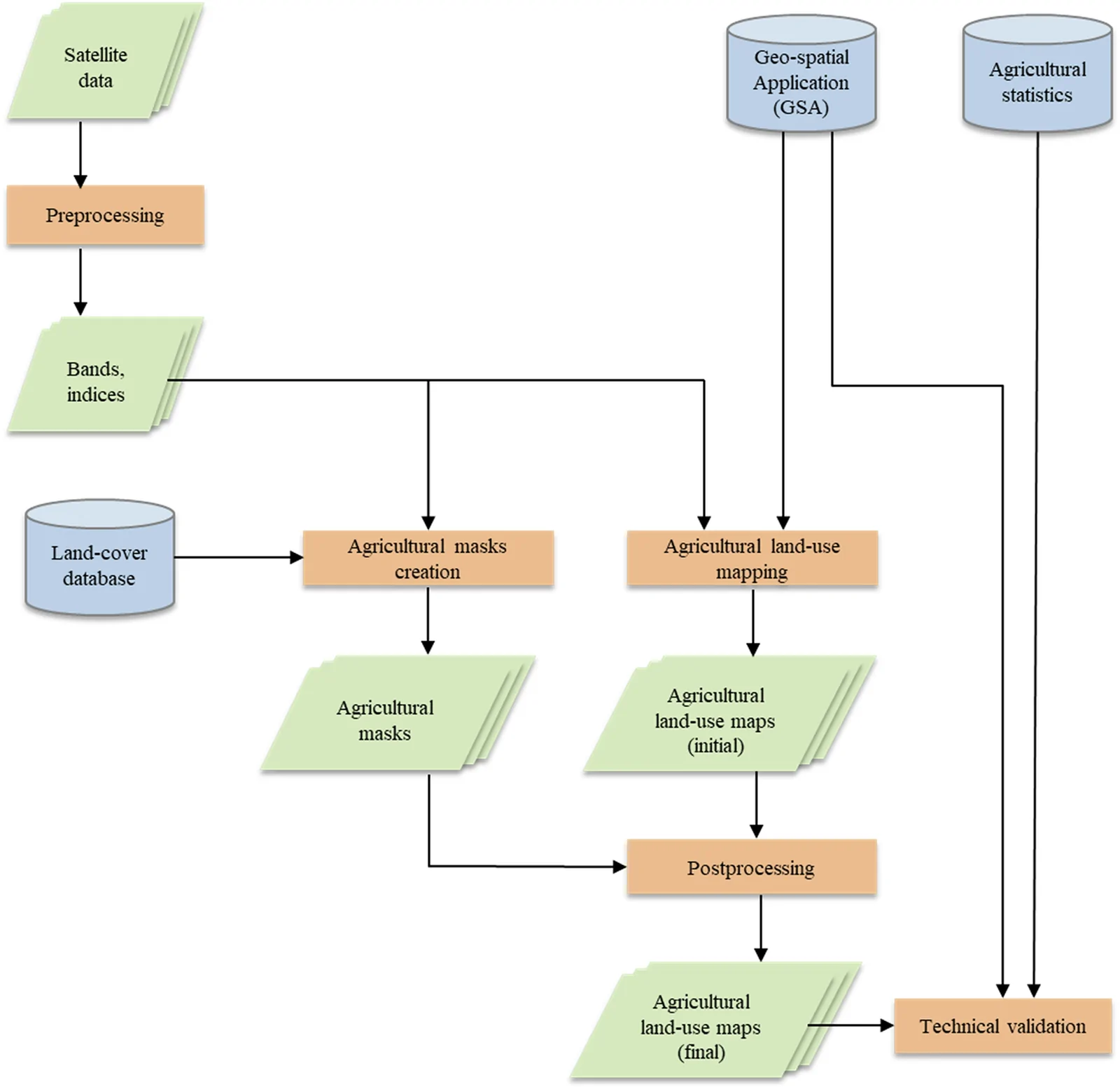
The general workflow we followed to create the agricultural land-use maps of Germany.

Fig 1 shows the general workflow we used in this study to create the agricultural LU maps. The main components of the workflow are satellite data preprocessing, agricultural LU mapping, agricultural masks creation, postprocessing of agricultural LU maps, and technical validation. Each component is explained in subsequent sections.

### Preprocessing of satellite data

In this study, all Level-1 (top-of-atmosphere) Landsat images (https://earthexplorer.usgs.gov) from 1990 to 2023 and Sentinel-2 images (https://dataspace.copernicus.eu) from 2015 to 2023 with cloud cover less than 75% were downloaded and preprocessed to generate analysis-ready data (ARD) of Germany using the Framework for Operational Radiometric Correction for Environmental Monitoring (FORCE)^40^. Generating the ARD images involved converting the top-of-atmosphere images to Level-2 (surface reflectance) images by correcting for radiometric, atmospheric, and geometric distortions^41–43^, masking clouds and cloud shadows using the Fmask algorithm^44–46^, and resampling of images to 30 m. Following the approach of Pham et al.^39^, we extracted the bands present in both sensors (red, green, blue, near-infrared, shortwave-infrared 1, shortwave-infrared 2) and generated three spectral indices, namely Normalized Difference Vegetation Index (NDVI), Normalized Difference Water Index (NDWI), and Soil-Adjusted Vegetation Index (SAVI). Consequently, each ARD image contained nine bands (six spectral bands and three spectral indices). An overview table showing the number of clear-sky observations (CSOs) generated from the ARD images per year, alongside the corresponding sensors, can be found in Table S1 of the Supplementary File.

### Agricultural land-use reference data

As reference data for agricultural LU mapping, we used the agricultural parcels declared by the EU farmers in the GSA as part of the Integrated Administration and Control System (IACS) to receive CAP payments. The data were obtained by the Thünen Institute, as a federal research institute, directly from the state-level paying agencies of the CAP subsidies in Germany. The coordinating body of the state-level paying agencies is the German Federal Ministry of Agriculture, Food, and Regional Identity (BMLEH) (https://www.bmleh.de/EN/Home/home_node.html).

**Fig. 2:**
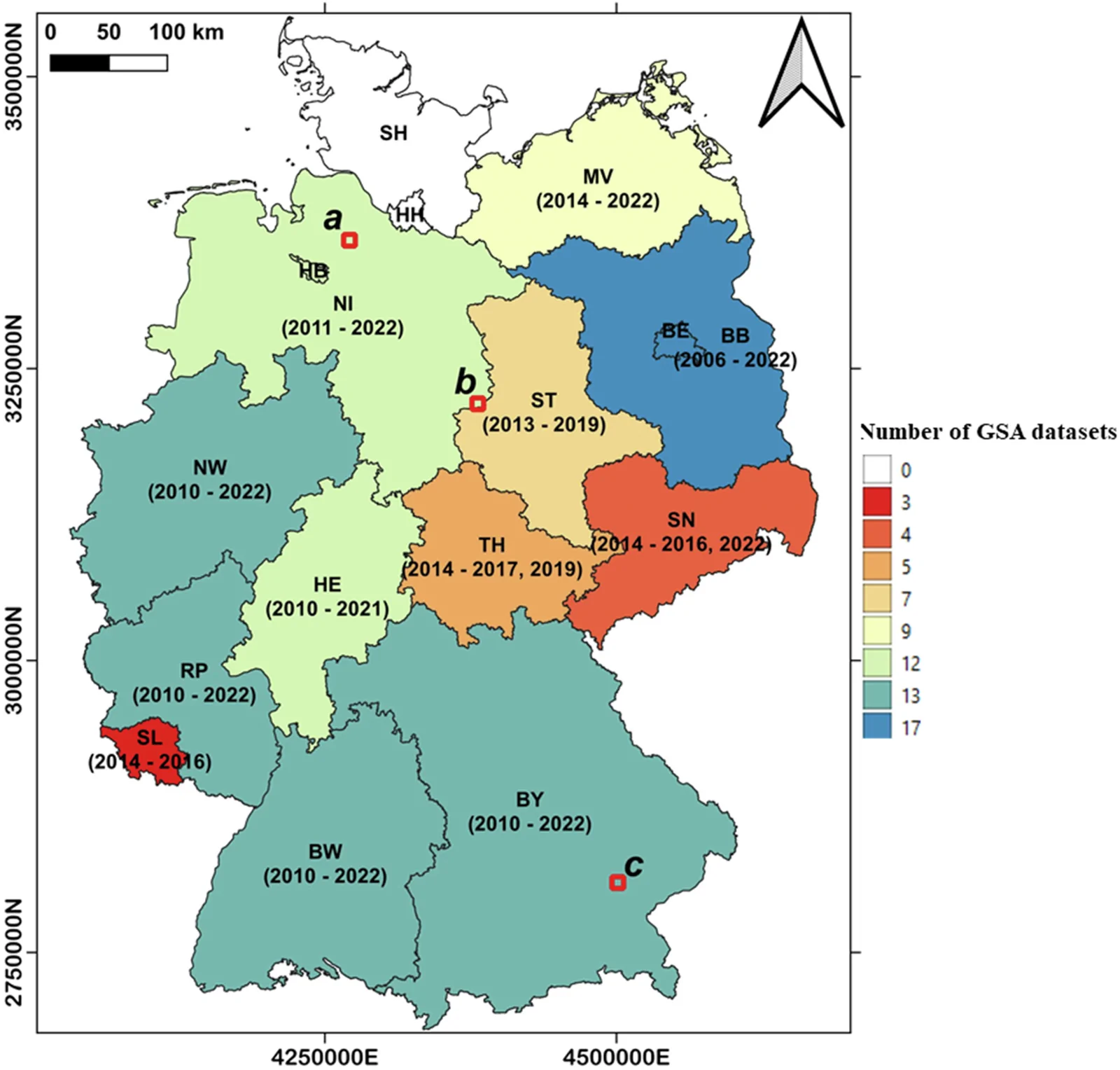
The number of available GSA datasets in Germany between 1990 and 2023. The years in which GSA datasets were available per federal state are in parentheses. Red boxes (**a**–**c**): see detailed views in Fig. 3. BW: Baden-Württemberg, BY: Bavaria, BE: Berlin, BB: Brandenburg, HB: Bremen, HH: Hamburg, HE: Hesse, NI: Lower Saxony, MV: Mecklenburg-Vorpommern, NW: North Rhine-Westphalia, RP: Rhineland-Palatinate, SL: Saarland, SN: Saxony, ST: Saxony-Anhalt, SH: Schleswig-Holstein, TH: Thuringia.

The GSA includes the spatial boundaries and LU types of all parcels for which subsidies were requested. The federal states in Germany are responsible for reviewing all GSA applications and subsequently providing the data for research and development based on individual data use agreements. Consequently, data availability varies between the federal states. The spatial and temporal coverage of the GSA data we had access to in Germany is shown in Fig. 2. Before 2006, GSA datasets were not available. The federal state of Brandenburg had the highest number of available GSA datasets from 2006 to 2022, while the federal state of Saarland had the lowest (2014–2016). No GSA data was available for Schleswig-Holstein and Hamburg. The GSAs for Bremen and Berlin are included in the datasets of Lower Saxony and Brandenburg, respectively.

In 2014, 2015, and 2016, the GSA had the highest spatial coverage (12 federal states). To ensure consistency in class definitions over time and with groups of classes used in the agricultural statistics of Germany, we extracted the main GSA classes and translated them into a condensed set of 14 classes: winter cereals, summer cereals, maize, grassland, potato, sugar beet, rapeseed, sunflower, legumes, horticultural crops, fallow land, vineyards, hops, and plantations. The three perennial classes (vineyards, hops, and plantations) were included to potentially identify the year of establishment. The translation table is in Table S2 of the Supplementary File.

### Agricultural land-use mapping

Given the non-availability of GSA before 2006 and the relatively low temporal density of ARD images before 2015, we opted for the mapping approach of Pham et al.^39^, which has been successfully used to map annual land cover and land use in the Baltic Sea region from 2000 to 2022^47^. This approach has three components: (1) the use of temporal encoding for generating annual time series data per pixel, (2) the application of two data augmentation techniques, namely random observation selection (ROS) and random day shifting (RDS), to simulate temporal patterns of data-sparse conditions and phenological variations, and (3) the use of a 1D-CNN model for training and prediction.

The temporal encoding involves generating arrays of 365 values per pixel and band, where each value represents the CSO for the day of year (DOY). Where no CSO is available for a particular DOY, zero is inserted into the array. The ROS involves randomly selecting proportions of the temporally encoded data for each reference sample during training, with the proportions ranging between 5% and 100%, to simulate data-sparse situations during model training. This is followed by RDS, which involves the random shifting of observations forward or backward within sixteen days, which corresponds to the temporal resolution of Landsat. This augmented time series, together with the class-wise reference labels, is finally passed to the 1D-CNN model for training. The 1D-CNN model comprises four convolution blocks. Each convolution block consists of two convolution layers, batch normalization, and rectified linear unit activation. Max-pooling is applied after the first, second, and third convolution blocks, while the output of the fourth convolution block is flattened and attached to a dense layer for prediction. For more details regarding the approach, readers are referred to Pham *et al*.^39^.

To train the model, we randomly sampled 10,000 pixels per class for each year with GSA data. Per year and class, the samples were split into 75% for training and 25% for validation. The respective training and validation samples per year were then merged to create a multi-year training and validation pool. The multi-year training samples were used to train the 1D-CNN model, while the multi-year validation samples were used to validate model performance during training. The model was trained for 100 epochs with a batch size of 512 and a learning rate of 0.001 on a computer with 48 GB of graphics processing unit (GPU) memory. After training, the model was applied to each ARD pixel per year to generate the initial agricultural LU maps of Germany from 1990 to 2023.

### Agricultural masks creation

The initial agricultural LU maps created in the previous section contain information for all pixel locations in Germany. To remove the non-agricultural pixels from those initial LU maps, we created annual agricultural masks. To create the annual agricultural masks, we first generated annual LC maps for Germany. As reference data for LC mapping, we used the German-wide reference database used for the calculation of GHG emissions in Germany by the Thünen Institute^48^. This reference database, which contains the main LC types of Germany, was created from a combination of LC datasets from the Coordination of Information on the Environment (CORINE) before 2000 and ATKIS thereafter. The CORINE dataset is accessible via the Copernicus Land Monitoring Service (https://land.copernicus.eu/en/products/corine-land-cover), and ATKIS was directly obtained by the Thünen Institute from the German Federal Agency for Cartography and Geodesy (https://bkg.bund.de). For our research, we had access to the LC samples in the reference database from 1990 to 2022.

Per year, we randomly sampled 1,500 pixels from the following LC classes: forest, cropland, perennial cropland, grassland, woody plants and hedges, wetlands, water, peat mining, built-up areas, and others. Those ten classes cover the entire surface area of Germany. The LC samples were split into 75% training and 25% validation per year and class, and subsequently merged to create a multi-year training and validation pool. We followed the same training procedure used for LU mapping and trained another 1D-CNN model using the multi-year pool of LC samples. The trained model was then applied annually to the ARD images to generate the annual LC maps.

To remove spatial noise, isolated misclassified pixels, and spurious LC transitions, we evaluated three spatiotemporal majority filters with the following dimensions (time, height, width): 3 × 3 × 3, 5 × 3 × 3, and 7 × 3 × 3 on the stack of LC maps from 1990 to 2023. The overall accuracy (OA) obtained for each filter across all years is shown in Fig. S1 of the Supplementary File. We proceeded with the filtered LC maps of the 7 × 3 × 3 filter, given that it resulted in the highest accuracies in all years. The annual agricultural masks were finally created by merging the cropland, perennial cropland, and grassland pixels from each filtered map into a mask layer per year. Compared with all agricultural pixels in the reference LC database that were neither used for training nor validation, the accuracies of the annual agricultural masks ranged between 92.15% (1990) and 93.22% (2020), as captured in Table S3 of the Supplementary File.

### Postprocessing of land-use maps

The initial agricultural LU maps underwent several postprocessing steps to derive the final maps. All pixels per LU map outside the agricultural mask of the corresponding year were removed. After the removal, a moving spatial majority filter (1 × 3 × 3) was applied to each LU map to remove isolated pixels. Given the relative stability of perennial crops (vineyards, hops, plantations) in Germany over time, a moving temporal majority filter (3 × 1 × 1) was applied to the spatially filtered LU maps centered on pixels classified as a perennial crop within 3-year windows to reinforce persistence. The final agricultural LU map per year was created by applying a sieve filter to remove objects smaller than 5 pixels (0.45 ha).

## Data Records

The datasets generated in this study are publicly available in this Zenodo repository^49^ (https://doi.org/10.5281/zenodo.20815677). This repository contains a zipped file called “HCTM_Data.zip”. Within this zipped file, there are two folders named “LU_Maps” and “CSO_Maps”. The “LU_Maps” folder comprises 34 cloud-optimized GeoTIFF (COG) images, corresponding to the agricultural LU maps from 1990 to 2023. Each image is named according to the format “HCTM_GER_[year]_rst_v101_COG.tif”. For each LU map, there is a corresponding clear-sky observation (CSO) map named “CSOS_GER_[year]_rst_v101_COG.tif” in the “CSO_Maps” folder. Each LU map contains a single band, and each pixel per band contains a positive integer value representing the agricultural LU type. Interpretation of the pixel values can be found in the color table files “HCTM_GER_LegendEN_rst_v101.clr” (English version) and “HCTM_GER_LegendDE_rst_v101.clr” (German version), which are located in the “LU_Maps” folder. In the color table, the first column represents the pixel value, the second to the fifth columns represent the RGBA color format, and the last column is the agricultural LU type. Each pixel in a CSO image contains 12 bands, representing the 12 months (January to December) of a year. Each CSO band contains the total number of CSOs per month of the year. All images are projected to the EPSG:3035 (ETRS89-extended/LAEA Europe) coordinate system, with a spatial resolution of 30 m.

## Data Overview

**Fig. 3:**
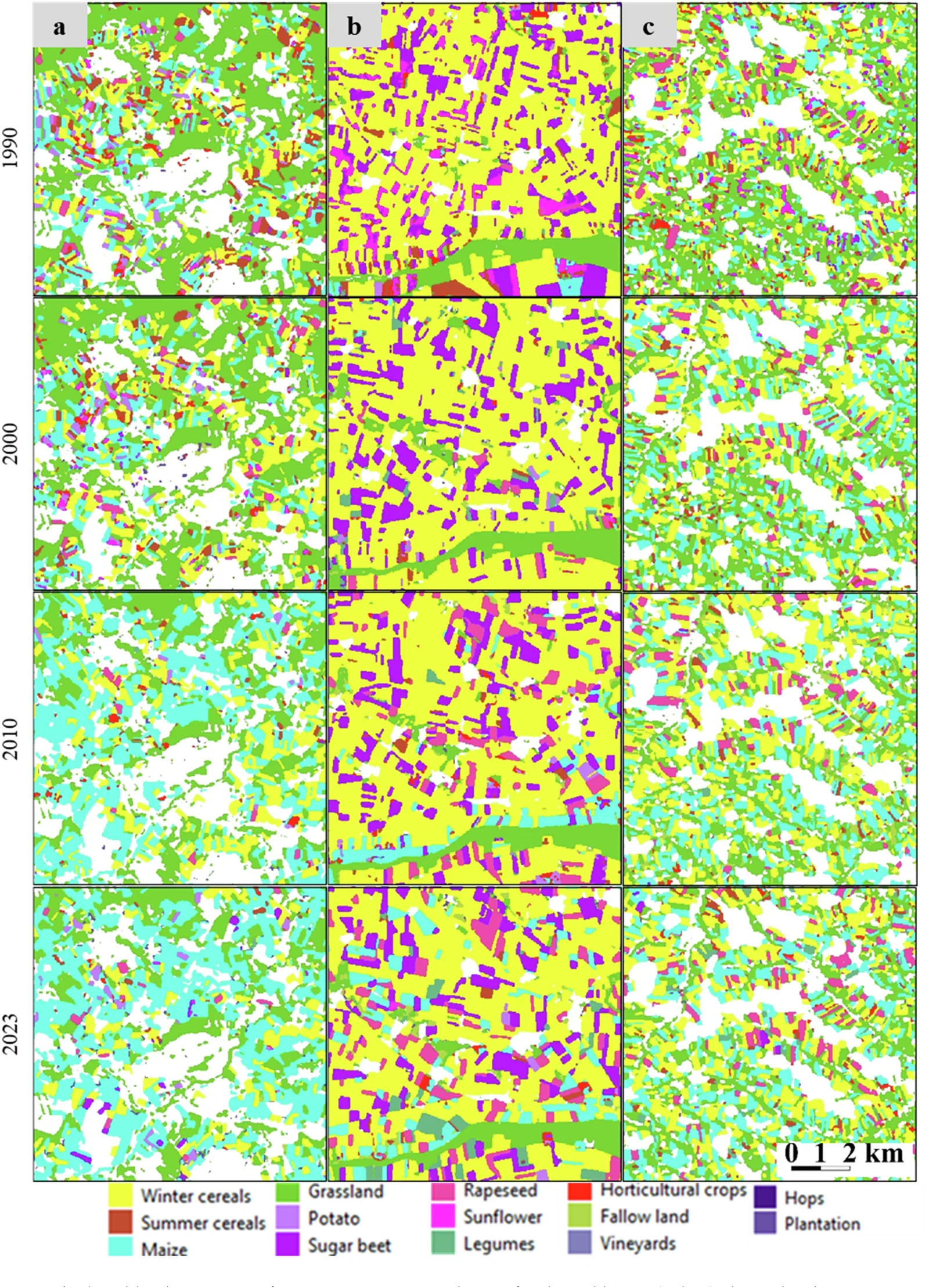
Agricultural land-use maps of 1990, 2000, 2010, and 2023 for the red boxes (**a**–**c**) shown in Fig. 2.

Fig 3 shows zoomed-in views of agricultural LU dynamics at the locations of the red boxes (a, b, c) previously shown in Fig. 2. Each box location corresponds to a 10 km^2^ grid. The grid in Fig. 3a is situated in the old drift morainic landscape of Lower Saxony, where the soil is sandy and less fertile. In both 1990 and 2000, grassland was the most dominant class. However, in both 2010 and 2023, many transitions from grassland to maize occurred, making maize the most dominant class. Similar results in Lower Saxony were observed by other researchers^28,29^. Those researchers assigned the German Renewable Energy Act (EEG), first introduced in 2000, as the main driver behind the transitions to maize.

In Fig. 3b, the grid borders Lower Saxony and Saxony-Anhalt, and falls within the loess and sandy loess landscape of Germany, where the soils are the most fertile, hence used more intensively for high-yield crops. Here, the LU types remained relatively stable over time, with winter cereals being the most dominant throughout the period. In Fig. 3c, the grid is located in the sheet gravel plains and tertiary hills of the Alpine Foreland in Bavaria, where the soils are moderately fertile. In 1990 and 2000, grassland had the highest share. Both 2010 and 2023 saw many grasslands transitioning to winter cereals, which then became the most dominant in both years. In summary, the data overview in Fig. 3 shows the usefulness of our LU maps in capturing long-term trends in agricultural LU and tracking LU changes over time.

**Fig. 4:**
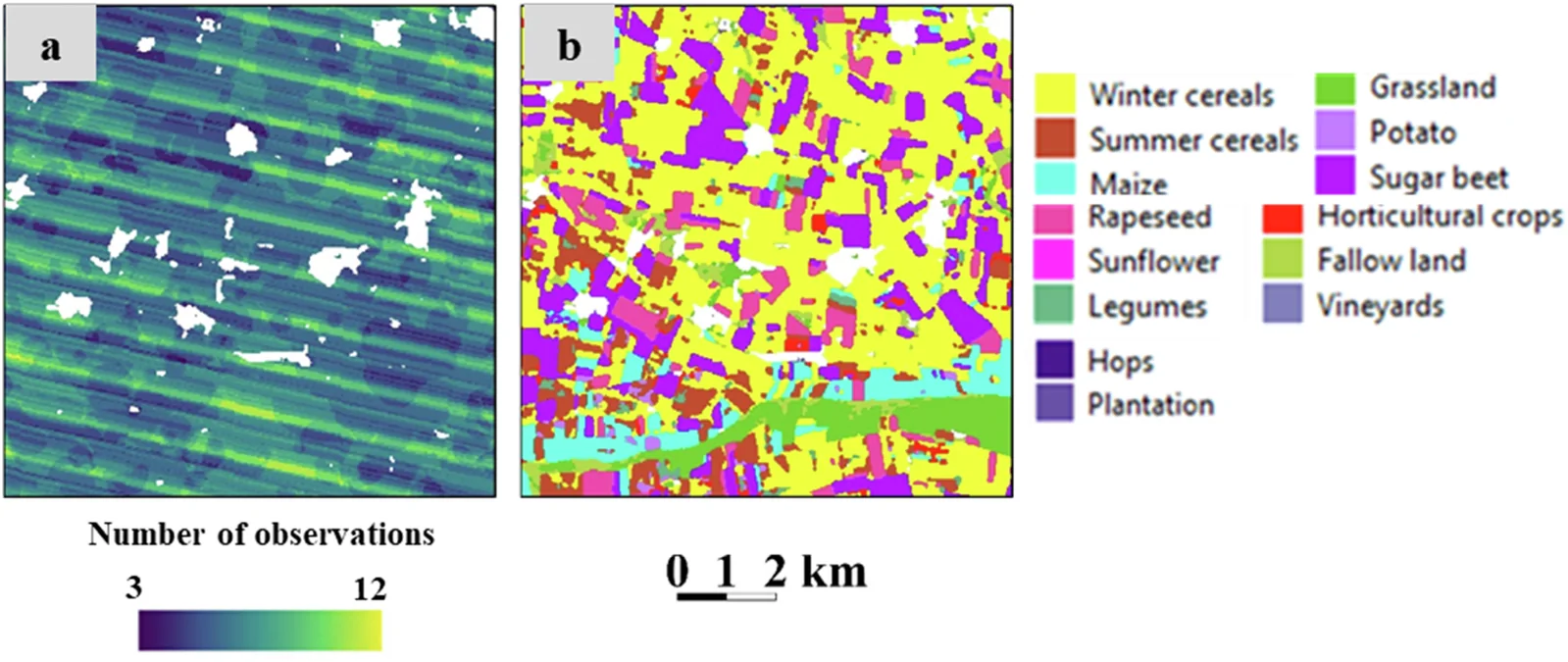
(**a**) The annual clear-sky observations (CSOs) in 2012 for the red box labelled *b* in Fig. 2. (**b**) The corresponding agricultural land-use map of 2012.

In 2003, the Scan Line Corrector (SLC) of Landsat 7 failed, resulting in about 22% of the pixels per scene not being scanned, thereby limiting the use of the data for scientific applications^50^. The limited use of the data is particularly crucial for 2012 because only Landsat 7 data was available in that year. Even though spatial artefacts (stripes) were visible in the annual CSO map in Fig. 4a, the corresponding LU map in Fig. 4b contains no spatial artefacts. The absence of spatial artefacts in our LU maps of 2012 aligns with the results reported by Pham et al.^39^ for the same year in Brandenburg, Germany.

## Technical Validation

### Overall accuracies

To assess the overall accuracy (OA) of each final agricultural LU map, we used all GSA pixels that were neither used for training nor validation to generate a confusion matrix per year (Figs. S2–S14 in the Supplementary File). We excluded the years from 2006 to 2010 in the accuracy assessment because the GSA was only available for Brandenburg, which does not cover all 14 classes. Based on each confusion matrix, we computed OA:

1 $${OA}=\frac{\mathop{\sum }\limits_{i=1}^{K}{{TP}}_{i}}{N}$$

where TP are the true positives, N is the total number of samples, and K is the number of classes. Table 1 presents the OA values and the corresponding median number of CSOs. Generally, the accuracies were high, ranging between 85% and 93%. The highest accuracy of 92.3% was achieved in 2018, and the lowest (85.3%) was in 2012. Accuracies above 90% were mostly achieved after 2015, when both Landsat and Sentinel images were available. The lowest median number of CSOs and the lowest OA were both recorded in 2012. The Pearson correlation coefficient (*r*) of 0.712 between the OA values and the median number of CSOs was statistically significant (p-value = 0.006), highlighting the impact of the number of observations on classification accuracy.

**Table 1:** Overall accuracy per year. The median CSOs were computed based on the reference areas per year.

Year	Overall accuracy	Median number of CSOs
2010	89.6%	11
2011	89.1%	17
2012	85.3%	6
2013	89.0%	11
2014	91.9%	15
2015	89.3%	23
2016	91.6%	29
2017	91.7%	28
2018	92.3%	54
2019	92.2%	46
2020	91.7%	53
2021	91.8%	37
2022	91.8%	61

### Class accuracies

**Fig. 5:**
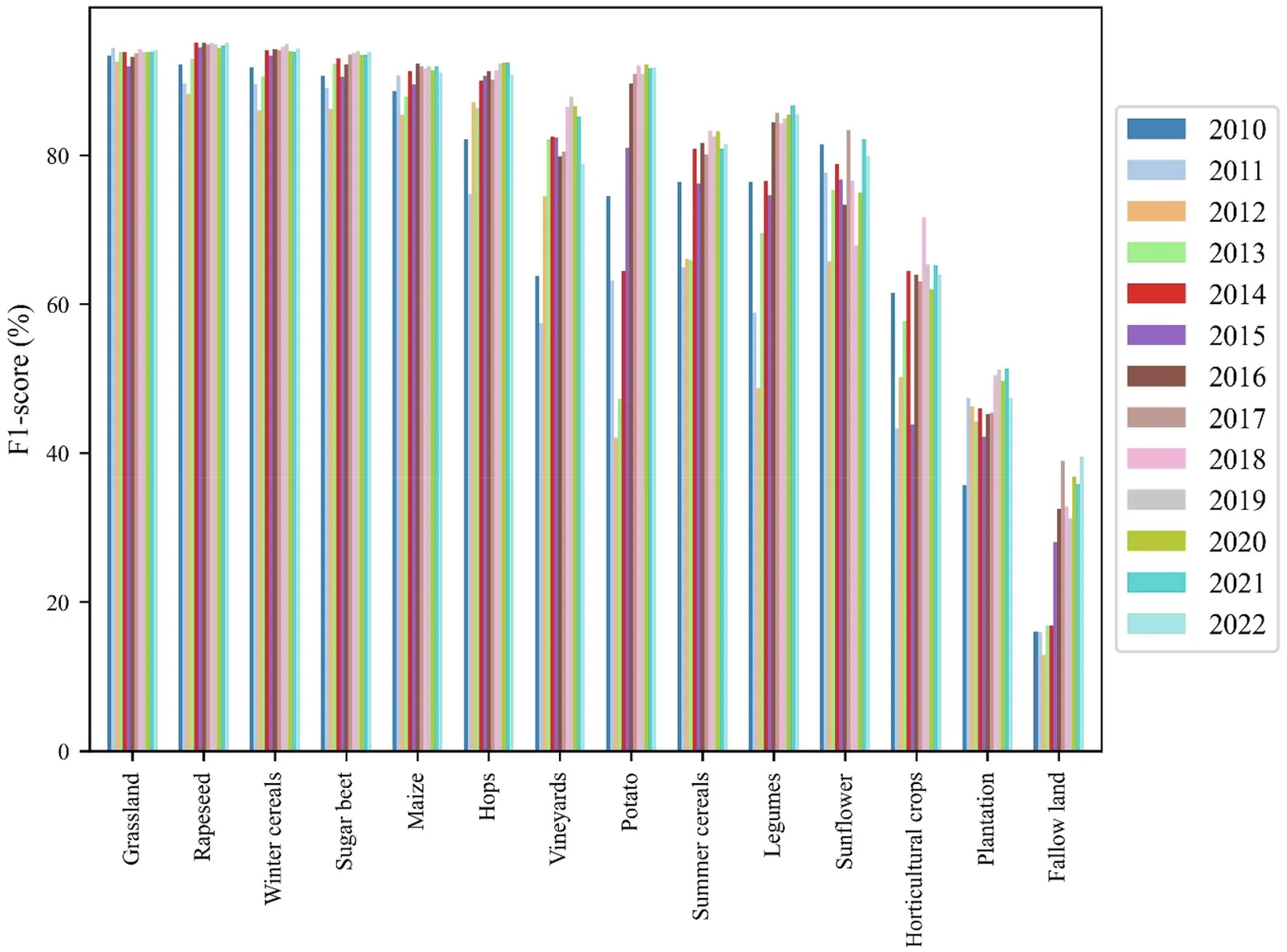
F1-scores for each agricultural LU type from 2010 to 2022.

To assess the accuracy of each class per year, we used each confusion matrix from 2010 to 2022 as the basis to calculate the F1-score, which is the harmonic mean of the user’s accuracy (UA) and producer’s accuracy (PA):

2 $${UA}=\frac{{TP}}{{TP}+{FP}}$$

3 $${PA}=\frac{{TP}}{{TP}+{FN}}$$

4 $$F1 \mbox{-} score=2\times \frac{UA\times PA}{UA+PA}$$

where TP, FP, and FN are the true positives, false positives, and false negatives, respectively. The F1-scores of each LU type corresponding to the years shown in Table 1 are depicted in Fig. 5.

When averaged over time, the highest accuracies (≥90%) were obtained by classes occupying high shares of agricultural land, like grassland, rapeseed, winter cereals, sugar beet, and maize. In comparison, classes such as fallow land and plantation, which occupy small shares of agricultural land, were predicted with the lowest accuracies (≤52%). Similar to the OA results, the lowest F1-scores were mostly recorded in 2012, while the highest were mostly obtained after 2015.

The lowest accuracy of grassland was 91.9% (2015), and the highest was 94.4% (2011). Rapeseed, winter cereals, sugar beet, and maize had accuracies greater than 85% in all years, with their lowest accuracies occurring in 2012. The highest accuracies of rapeseed (95.1%) and maize (92.3%) both occurred in 2016, while those of winter cereals (94.9%) and sugar beet (93.9%) both occurred in 2019. Hops had accuracies ranging from 74.8% (2011) to 92.4% (2020). Similar to hops, vineyards were at their lowest (57.5%) in 2011, but the highest (87.9%) was in 2019. In 2012, the potato class registered its lowest accuracy (42.0%), with its highest (92.2%) in 2020. Indeed, the potato class had the highest disparity between its lowest and highest accuracies. Summer cereals were detected at the lowest accuracy (65.0%) in 2011 and the highest (83.3%) in 2018. Legumes were classified with accuracies ranging from 48.7% (2012) and 86.7% (2021). Sunflower accuracies were between 65.7% (2012) and 83.3% (2017). The lowest accuracy of horticultural crops was 43.2% in 2011, and the highest was 71.6% in 2018. Plantation had accuracies ranging from 35.7% (2010) to 51.3% (2021). Overall, the lowest accuracies were recorded for fallow land, ranging from 12.9% (2012) to 39.5% (2022), making it the most difficult class to predict.

**Fig. 6:**
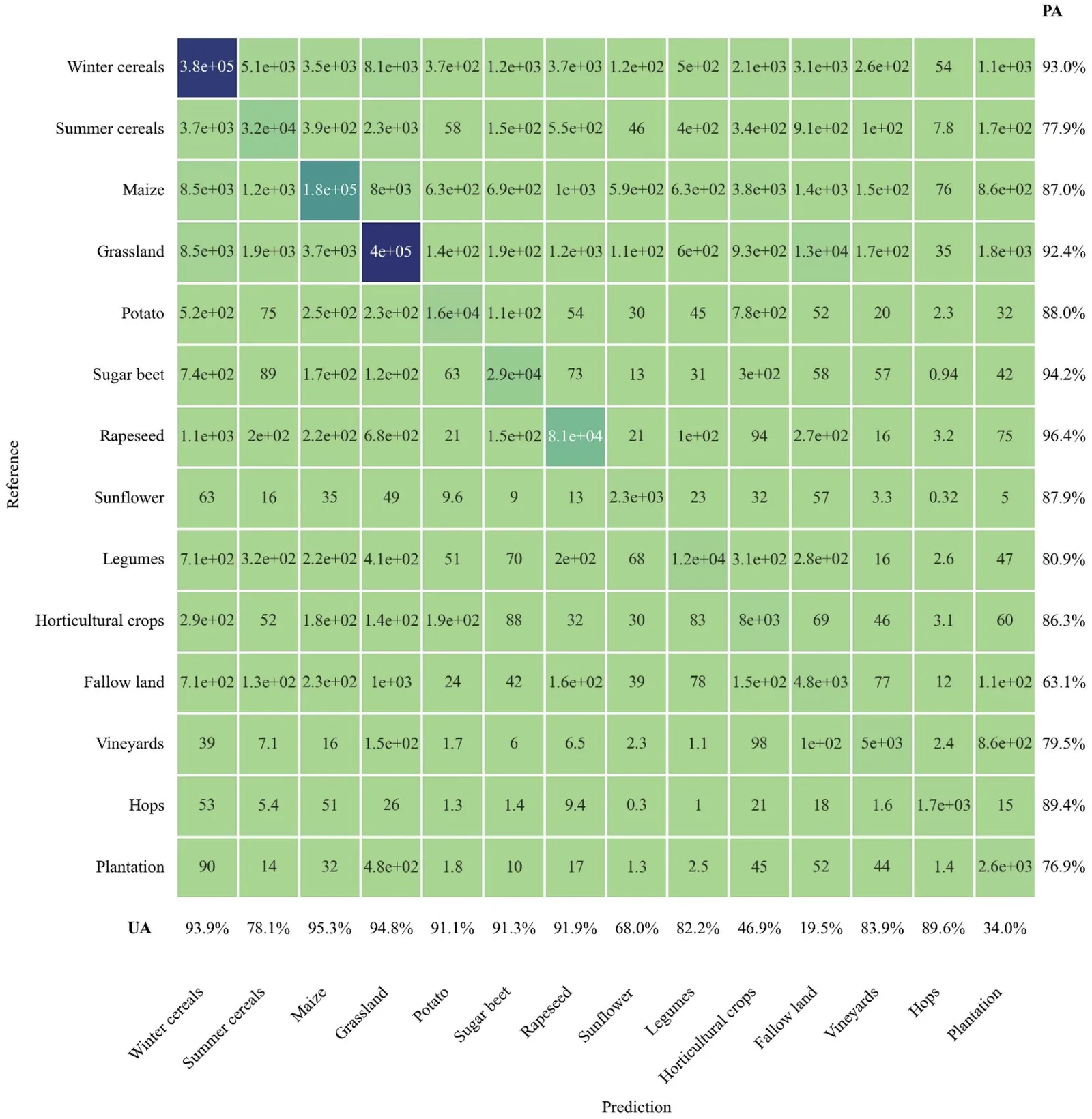
The composite confusion matrix generated from all yearly confusion matrices from 2010 to 2022 (Figs. S2–S14 in the Supplementary File). The matrix entries are areas (km^2^).

For a one-shot overview of the confusion between the classes over time, we used the yearly confusion matrices to create a composite confusion matrix (Fig. 6). Among the best-performing classes, rapeseed and sugar beet achieved very high producers’ accuracies (PA = 96.4% and 94.2%, respectively), indicating low omission errors and strong separability from other crops. Winter cereals (PA = 93.0%) and grassland (PA = 92.4%) also exhibited high detection rates. Users’ accuracies for these classes were similarly high (UA > 91% for rapeseed, sugar beet, winter cereals, maize, potato, and grassland), demonstrating low commission errors and high thematic reliability of the mapped categories.

Maize, potato, sunflower, horticultural crops, and hops showed good to moderate producers’ accuracies (PA between 86% and 89%), although sunflower exhibited a comparatively low user’s accuracy (UA = 68.0%), indicating notable commission errors. Legumes, vineyards, summer cereals, and plantations reached moderate producers’ accuracies (PA between 76% and 81%), suggesting increased omission errors, likely due to spectral and phenological similarities with other LU types.

Fallow land was the weakest-performing class, with a low producer’s accuracy (PA = 63.1%) and particularly low user’s accuracy (UA = 19.5%). This indicates both substantial omission and commission errors and highlights the difficulty in particularly distinguishing fallow land from grasslands. The substantial confusion between fallow land and grassland could be attributed to how fallow land is represented in the GSA. In the GSA, fallow lands are not just bare lands, but they are sometimes covered with grass. In such instances, fallow lands and grasslands are spectrally and phenologically similar, which possibly explains the high confusion between them. Similar to fallow lands, plantation (UA = 34.0%) and horticultural crops (UA = 46.9%) exhibited low user accuracies, suggesting that these mapped classes contain considerable proportions of misclassified pixels. Low accuracies for plantation and horticultural crops were also reported in other studies^15,18^. Preidl et al.^18^ assigned the low accuracy of plantations to their species composition and spectral similarity with grassland. Regarding horticultural crops, the class is broad, and it contains several crops, such as strawberries and asparagus. Both strawberries and asparagus were among the classes mapped with the lowest accuracies by Preidl et al.^18^. The authors attributed this low accuracy to the fact that asparagus and strawberries are usually grown on fields that are barely larger than a Sentinel-2 pixel, leading to spectral mixing with adjacent LU.

Generally, the dominant misclassification patterns revealed three main confusion groups. First, mutual confusion within the broad cereal group (winter cereals, summer cereals, and maize) was evident. Second, a confusion within the semi-natural vegetation group was noticed, characterized by strong confusion between grassland and fallow land, and to a lesser extent, plantation. Third, confusion among intensively managed specialty crops was observed, particularly between potato, horticultural crops, and sugar beet. Although our class definitions and thematic details differ, these three groups of confusion patterns were largely present in the maps of Blickensdörfer et al.^15^ and Griffiths et al.^17^. For example, Griffiths et al.^17^ reported confusions between winter and spring cereals, while Blickensdörfer et al.^15^ observed substantial confusions within cereal groups and between different cereal subclasses. Blickensdörfer et al.^15^ reported confusions between grassland and perennial vegetation classes, such as small woody features and orchards (plantations), while Griffiths et al.^17^ similarly highlighted the difficulties in separating grassland from vineyards. Blickensdörfer et al.^15^ reported confusion among specialty crops like horticultural crops (asparagus, onion, and other vegetables) and potato. These confusion patterns likely reflect overlapping phenological trajectories, similar canopy structures, and management practices that are difficult to disentangle using spectral time series alone.

In summary, the classification performs robustly for major LU types with distinct phenological signatures. Classes characterized by heterogeneous management or transitional vegetation states (e.g., fallow land, horticultural crops, and plantation) remain challenging. However, those classes cover very small shares of agricultural land in Germany. For further quantitative analyses using our products, those classes should be handled with caution.

### Comparison with official statistics

**Fig. 7:**
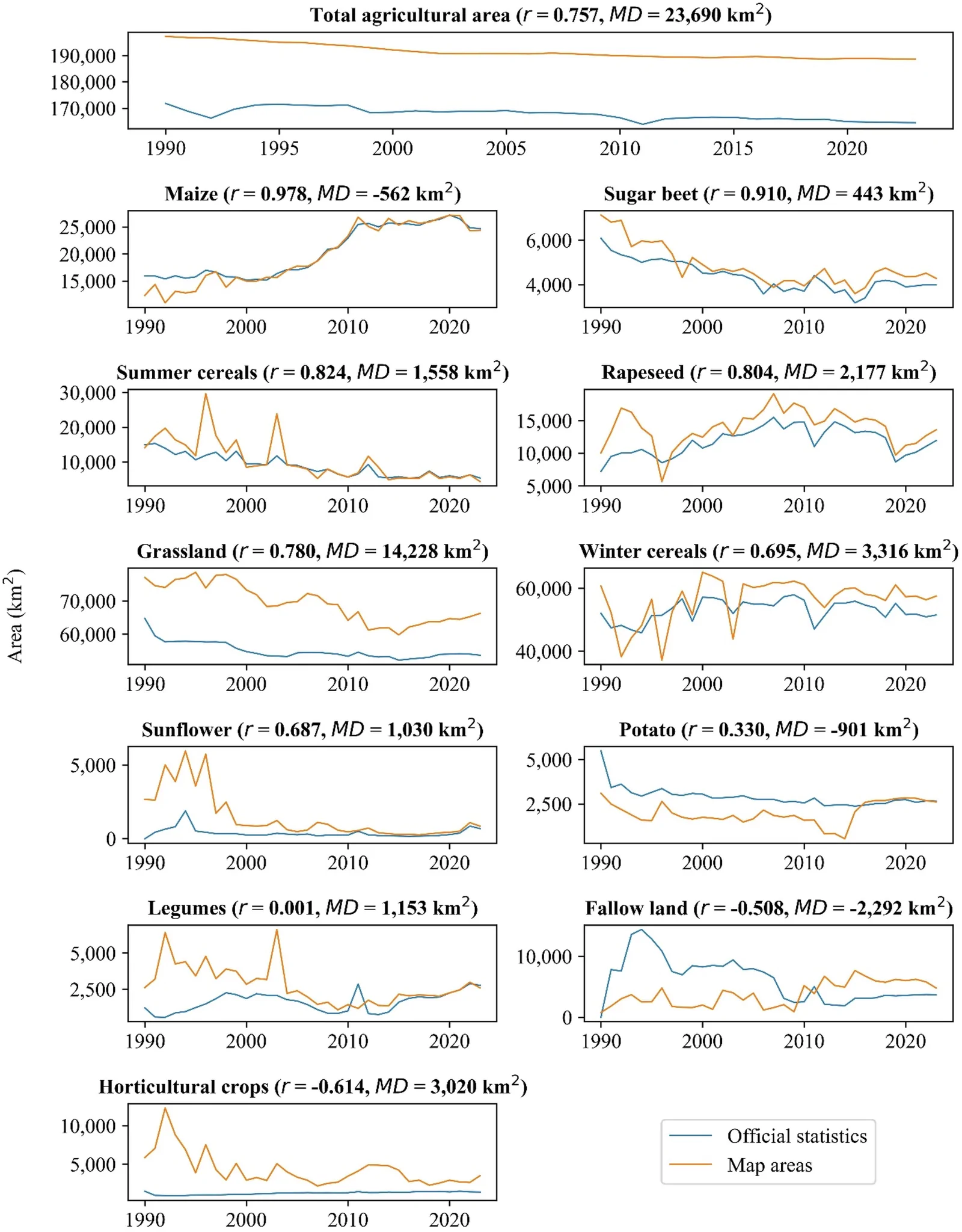
Comparison of map areas with official statistics. In all plots, *r* is the Pearson correlation coefficient, and *MD* is the mean deviation between the map areas and the official statistics.

To further assess the plausibility of our maps, we compared our map areas with the corresponding areas in the official statistics^8^. Although areas directly derived from our classification maps via pixel counting are biased estimates due to classification errors and do not serve as direct proxies for agricultural statistics, our overarching goal was to assess how those biased map area estimates compare with official statistics, given the absence of yearly reference samples to be used as basis for generating unbiased area estimates from classification maps following the approach of Olofsson et al.^51^. For the comparison, we calculated the Pearson correlation coefficient (*r*) and mean deviation (*MD*) between our map areas and the official statistics. With the Pearson correlation coefficient, we measured the strength and direction of the linear relationship between our map areas and those of the official statistics. The *MD*, which was calculated as the mean of the yearly differences between the map areas and official statistics, was used to assess the level of overestimation or underestimation in our map areas over time. Fig 7 shows the comparison between the map areas (orange lines) and official statistics (blue lines) for the total agricultural area, followed by the most dominant LU classes. The classes have been ordered by their respective Pearson correlation coefficients.

Regarding the total agricultural area, a high positive correlation (*r* = 0.757) was observed, with a tendency towards overestimation in the map areas for all years. This overestimation can be explained by the fact that in our agricultural LU maps, we map all agricultural areas in the open landscape, whereas the official statistics only account for land that is officially used for agricultural production.

Eight classes (maize, sugar beet, summer cereals, rapeseed, grassland, winter cereals, sunflower, potato) showed positive correlations, while mapped legume areas did not correlate with the official statistics. The remaining classes (fallow land, horticultural crops) exhibited negative correlations, confirming the difficulty in accurately predicting them. Fallow lands were often mistaken for grasslands due to similar spectral profiles, thereby increasing uncertainty in their prediction. Horticultural crops, which are usually grown on fields barely larger than a Sentinel-2 pixel^18^, often spectrally mix with adjacent LU classes, making their prediction difficult.

Maize and sugar beet had very high correlations (*r* > 0.9), with maize being underestimated and sugar beet being overestimated. Summer cereals, rapeseed, grassland, winter cereals, and sunflower had high correlations (0.6 < *r* ≤ 0.83), and all of them were overestimated. Potatoes, which were underestimated, had a moderate correlation (0.33). Legumes were overestimated and exhibited a weak relationship (*r* = 0.001). Although both fallow land and horticultural crops obtained negative correlations, fallow land tended to be underestimated, while horticultural crops were overestimated. Overall, sugar beet obtained the smallest deviation from the official statistics over time.

## Usage Notes

### Ready-to-use products

The provided LU maps in the COG format enable data hosting in Hypertext Transfer Protocol (HTTP) servers and efficient data handling in cloud computing environments. Together with the provided color table, the maps can be visualized in various geospatial software, such as QGIS and ArcGIS.

### Comparison with existing products

Several factors limit a direct comparison of our maps with other existing German-wide maps. Firstly, all the maps have different class catalogues and sometimes different class definitions. In our research, we mapped broader classes such as winter cereals and summer cereals. In all the other existing studies, except for Griffiths et al.^17^, finer classes within winter and summer cereals, such as winter wheat and spring wheat, were mapped. Secondly, the accuracy assessment in the other studies used only a few pixels for map validation. In our study, all non-training pixels were used for validation. Thirdly, the spatial resolution (30 m) of our products is lower than that of the existing products, except Griffiths et al.^17^, which has the same spatial resolution as ours. Given those three reasons, the comparison here should be considered as indicative and not conclusive.

We compared the F1-scores of our products with those directly reported in these three existing studies^15–17^. Where possible, we mapped their classes to our 14 classes to enable the comparison. Figs. S15–S21 in the Supplementary File show the comparisons. In 2016, we obtained higher accuracies than Griffiths et al.^17^ for all classes mapped by both products. In 2017, the F1-scores of Blickensdörfer et al.^15^ were higher regarding sugar beet and vineyards, equal regarding rapeseed, and lower regarding the other classes. Compared with Gessner et al.^16^, we obtained higher accuracies for grassland, winter cereals, maize, and summer cereals in all years from 2018 to 2022, higher accuracy for sugarbeet in 2018, 2019, and 2022, higher accuracy for potato in 2018 and 2019, and higher accuracy for legumes in 2022. For the remaining classes from 2018 to 2022, our accuracy was lower, with plantation being the lowest. In all years, fallow land was not mapped in any of the existing studies we evaluated.

### Using our products for further analysis

The distinct advantage of our LU map time series is the ability to facilitate spatially explicit analysis of agricultural LU changes over time that cannot be achieved with aggregated data, such as agricultural statistics. Although our map areas generally aligned well with official statistics, the areas extracted from our maps are not agricultural statistics and are therefore not meant to replace official agricultural statistics. To ensure accurate estimation of areas from classification maps, it has been recommended in the literature^51^ to combine map-based areas with reference-based statistics. For accurate estimation of areas from classification maps, readers are referred to Chapters 23–26 of the Handbook on Remote Sensing for Agricultural Statistics (https://fao-eostat.github.io/UN-Handbook/). With correctly estimated areas, our LU maps could be used to spatially and temporally disaggregate agricultural statistics.

The impact of the number of image observations on crop mapping accuracy is well documented in the literature^35,36,52^. Table 1 revealed the significant correlation between the number of CSOs and OAs, which was largely corroborated by the work of Pham et al.^39^. This indicates a general problem of transferring models trained in data-rich years to data-sparse years. It is important to add the caveat that a high number of CSOs does not guarantee a high classification accuracy, and a low number of CSOs does not necessarily lead to a lower classification accuracy. The actual number of CSOs, as well as the distribution of the CSOs throughout the season, may have varying impacts on different LU types. Nevertheless, given the significant correlation between the CSOs and OAs as shown in Table 1, the main uncertainty in our products could be tied to the number of CSOs per pixel. Therefore, when using our maps for further analysis, uncertainty modelling ought to be considered. In the absence of yearly reference samples to create spatially explicit uncertainty maps, observation uncertainty maps could be generated from the provided CSO layers and used as practical proxies for spatial uncertainty. An observation uncertainty map per year could be created using the following logic: (1) pixels with CSOs available throughout most or all months would have low uncertainty values, indicating high temporal support for accurately capturing LU phenological patterns; (2) pixels with CSOs in only a few months would be assigned higher uncertainty values, reflecting limited temporal information and a greater potential for classification uncertainty; (3) pixels without any clear-sky observations would be assigned the highest uncertainty values.

The application of the 3-year temporal persistence constraint to vineyards, hops, and plantations was geared at reducing isolated year-to-year fluctuations that were unlikely to represent actual LU changes. This assumption is justified by the perennial nature of these crop types, which typically remain under the same management for many years in Germany. However, this temporal filtering introduces a trade-off between reducing classification noise and preserving the exact timing of LU transitions. In rare cases, rapid establishment or removal of perennial systems occurring within a short time span (e.g., one or two years) may be suppressed, potentially leading to an underestimation of short-term transitions. Consequently, studies focusing on precise annual change detection should consider the potential effects of this temporal smoothing in our products.

### Limitations in our accuracy assessment

The training samples and the remaining non-training pixels used to assess the accuracy of the LU maps both originate from the same GSA dataset; hence, they are not truly independent in terms of data source or annotation methodology. Additionally, some of the remaining GSA pixels used for the accuracy assessment could also originate from agricultural fields already represented in the training data. This spatial dependence could potentially inflate the reported accuracies because pixels within the same agricultural field generally have highly similar spectral-temporal characteristics. Consequently, the reported accuracies in this paper may represent an optimistic upper bound of model performance. Therefore, users with independent validation samples are encouraged to validate the maps with their data and check conformance with our reported accuracies.

Due to the non-availability of reference data before 2006 for model training and validation, the LU maps before 2006 may contain higher levels of uncertainty, which we were unable to quantify in this research through accuracy assessment. However, Pham et al.^39^ showed that their approach, which we adopted in this study, could be applied to images from 2010 to 2021, even though it was trained only on data from 2018. Indeed, a visual assessment of the maps (e.g., for 1990 and 2000 in Fig. 3) reveals the spatial consistency of the LU maps, even before 2006. Further, comparing our map areas with the official statistics revealed similar trends for the most dominant LU types, even before 2006, indicating a plausible temporal behavior in years without reference data. Irrespective of this acceptable performance, caution should still be exercised when using the maps before 2006, and the joint use with the provided CSO layers, as additional information on satellite data availability, is highly recommended.

### Training data reusability and temporal transfer

An important limitation of the current workflow is that reference data scarcity was addressed primarily through model-side augmentation strategies rather than explicit training-data transfer across years. Some studies have demonstrated the potential of domain adaptation methods to transfer classifiers between source and target domains by aligning feature distributions or iteratively incorporating pseudo-labelled target samples^53,54^. Likewise, temporal sample propagation approaches can generate training data for historical periods by identifying stable pixels and removing locations that exhibit land-cover change^55^. These approaches may be particularly valuable for extending LU mapping to years and regions with limited reference data availability. Future work would investigate the integration of such methods with the present temporal deep-learning framework to improve the exploitation of historical reference information and further reduce dependence on contemporaneous training samples.

## Supplementary information

Supplementary information

## Data Availability

All the generated maps in this study are publicly available in this Zenodo repository^49^ (https://doi.org/10.5281/zenodo.20815677). This repository contains a zipped file called “HCTM_Data.zip”. Within this zipped file, there are two folders named “LU_Maps” and “CSO_Maps”. The “LU_Maps” folder comprises 34 cloud-optimized GeoTIFF (COG) images, corresponding to the agricultural LU maps from 1990 to 2023, and two legend files in English (HCTM_GER_LegendEN_rst_v101.clr) and German (HCTM_GER_LegendDE_rst_v101.clr). The “CSO_Maps” folder contains corresponding clear-sky observation (CSO) maps named “CSOS_GER_[year]_rst_v101_COG.tif”. The maps are provided in the EPSG:3035 coordinate reference system at a 30 m spatial resolution.

## Code availability

The pseudocode for model training (Pseudocode 1), model inference (Pseudocode 2), and the code for the model architecture (Code 1) are provided in the Supplementary File. The preprocessing of the Landsat and Sentinel images to create the ARD images was done with FORCE (https://github.com/davidfrantz/force) using the parameter file (Parameter File 1) located in the Supplementary File. Generating and analyzing the maps was done in Python 3.10.12 using several libraries, including NumPy 1.26.4, GDAL 3.6.2, Rasterio 1.4.3, and TensorFlow 2.20.0. Visualization of the preprocessed images and predicted LU maps was done in QGIS 3.4.0 LTR.
