# Supplementary material for: Nationwide annual agricultural land-use maps of Germany from 1990 to 2023 derived from satellite imagery
Source: Sci Data. 2026 Sep 26;13:1353. doi: 10.1038/s41597-026-08375-w (PMC13615969; doi:10.1038/s41597-026-08375-w)
Supplement: Supplementary file 1 [file 41597_2026_8375_MOESM1_ESM.docx]

Supplementary File:

**Nationwide annual agricultural land-use maps of Germany from 1990 to 2023 derived from satellite imagery**

Gideon Okpoti Tetteh ^1,*^, Vu-Dong Pham ^3,4^, Marcel Schwieder ^1,2^, Lukas Blickensdörfer ^1,2^, Alexander Gocht ^1,5^, Sebastian van der Linden ^3,4^, Stefan Erasmi ^1^

^1^ Thünen Institute of Farm Economics, Bundesallee 63, 38116 Braunschweig, Germany

^2^ Geography Department, Humboldt University of Berlin, Unter den Linden 6, 10099 Berlin, Germany

^3^ Earth Observation and Geoinformation Science Lab, Institute of Geography and Geology, University of Greifswald, Friedrich-Ludwig-Jahn-Str. 16, 17489 Greifswald, Germany

^4^ Interdisciplinary Centre for Baltic Sea Region Research (IFZO), University of Greifswald, 17489 Greifswald, Germany

^5^ Agricultural Development and Trade Group, Department of Agricultural Economics, Humboldt University of Berlin, Unter den Linden 6, 10099 Berlin, Germany

* Corresponding author. Email address: [gideon.tetteh@thuenen.de](mailto:gideon.tetteh@thuenen.de)

Table S1. Overview of the number of clear-sky observations and the corresponding sensors per year.

| **Year** | **Minimum** | **Maximum** | **Mean** | **Median** | **Sensors** |
| --- | --- | --- | --- | --- | --- |
| 1990 | 0 | 25 | 9.90 | 10 | Landsat 5 |
| 1991 | 0 | 20 | 9.09 | 9 | Landsat 5 |
| 1992 | 0 | 22 | 9.35 | 9 | Landsat 5 |
| 1993 | 0 | 20 | 6.59 | 7 | Landsat 5 |
| 1994 | 0 | 19 | 8.02 | 8 | Landsat 5 |
| 1995 | 0 | 17 | 7.63 | 8 | Landsat 5 |
| 1996 | 0 | 18 | 6.79 | 7 | Landsat 5 |
| 1997 | 0 | 17 | 7.75 | 8 | Landsat 5 |
| 1998 | 0 | 19 | 6.36 | 6 | Landsat 5 |
| 1999 | 0 | 28 | 13.12 | 13 | Landsat 5, Landsat 7 |
| 2000 | 0 | 27 | 12.00 | 12 | Landsat 5, Landsat 7 |
| 2001 | 0 | 25 | 11.29 | 11 | Landsat 5, Landsat 7 |
| 2002 | 0 | 29 | 10.87 | 11 | Landsat 5, Landsat 7 |
| 2003 | 0 | 28 | 12.58 | 13 | Landsat 5, Landsat 7 |
| 2004 | 0 | 26 | 11.78 | 12 | Landsat 5, Landsat 7 |
| 2005 | 0 | 36 | 14.81 | 15 | Landsat 5, Landsat 7 |
| 2006 | 0 | 29 | 14.57 | 15 | Landsat 5, Landsat 7 |
| 2007 | 0 | 27 | 9.08 | 9 | Landsat 5, Landsat 7 |
| 2008 | 0 | 23 | 9.01 | 9 | Landsat 5, Landsat 7 |
| 2009 | 0 | 30 | 11.98 | 12 | Landsat 5, Landsat 7 |
| 2010 | 0 | 25 | 10.34 | 10 | Landsat 5, Landsat 7 |
| 2011 | 0 | 35 | 15.57 | 15 | Landsat 5, Landsat 7 |
| 2012 | 0 | 20 | 5.79 | 6 | Landsat 7 |
| 2013 | 0 | 30 | 11.15 | 11 | Landsat 7, Landsat 8 |
| 2014 | 0 | 34 | 15.42 | 16 | Landsat 7, Landsat 8 |
| 2015 | 0 | 51 | 21.60 | 21 | Landsat 7, Landsat 8, Sentinel-2A |
| 2016 | 0 | 59 | 28.58 | 29 | Landsat 7, Landsat 8, Sentinel-2A |
| 2017 | 0 | 68 | 28.36 | 27 | Landsat 7, Landsat 8, Sentinel-2A, Sentinel-2B |
| 2018 | 1 | 96 | 52.64 | 54 | Landsat 7, Landsat 8, Sentinel-2A, Sentinel-2B |
| 2019 | 1 | 85 | 46.05 | 46 | Landsat 7, Landsat 8, Sentinel-2A, Sentinel-2B |
| 2020 | 1 | 99 | 51.25 | 52 | Landsat 7, Landsat 8, Sentinel-2A, Sentinel-2B |
| 2021 | 0 | 79 | 37.05 | 37 | Landsat 7, Landsat 8, Sentinel-2A, Sentinel-2B |
| 2022 | 1 | 112 | 59.22 | 60 | Landsat 7, Landsat 8, Landsat 9, Sentinel-2A, Sentinel-2B |
| 2023 | 0 | 88 | 47.36 | 48 | Landsat 7, Landsat 8, Landsat 9, Sentinel-2A, Sentinel-2B |

Overall accuracy (OA)

|  | unfiltered | 3x3x3 | 5x3x3 | 7x3x3 |
| --- | --- | --- | --- | --- |
| 1990 | 74.9505% | 79.5878% | 79.936% | 80.1741% |
| 1991 | 74.3883% | 80.0351% | 80.2753% | 80.6174% |
| 1992 | 73.3356% | 79.7978% | 80.7216% | 80.9364% |
| 1993 | 72.1908% | 79.9875% | 80.7559% | 81.1307% |
| 1994 | 75.3876% | 80.1834% | 80.718% | 81.102% |
| 1995 | 74.7208% | 80.2861% | 80.7324% | 81.0165% |
| 1996 | 73.7117% | 80.199% | 80.7313% | 81.0699% |
| 1997 | 75.1105% | 79.9913% | 80.7564% | 81.1589% |
| 1998 | 73.167% | 80.3476% | 80.8563% | 81.1497% |
| 1999 | 76.7343% | 80.5212% | 80.9516% | 81.1485% |
| 2000 | 76.6845% | 80.698% | 80.9293% | 81.2201% |
| 2001 | 76.9876% | 80.6594% | 81.1923% | 81.3485% |
| 2002 | 76.2357% | 80.9299% | 81.2844% | 81.5855% |
| 2003 | 76.8989% | 81.0587% | 81.5487% | 81.8209% |
| 2004 | 76.835% | 81.4567% | 81.8523% | 81.9949% |
| 2005 | 78.3486% | 81.765% | 82.0417% | 82.2158% |
| 2006 | 78.6562% | 81.8094% | 82.0837% | 82.3682% |
| 2007 | 76.2614% | 81.7735% | 82.2743% | 82.4478% |
| 2008 | 77.0583% | 81.7363% | 82.3406% | 82.5735% |
| 2009 | 78.4806% | 81.9879% | 82.2835% | 82.4839% |
| 2010 | 78.0717% | 82.1418% | 82.2486% | 82.5355% |
| 2011 | 78.3663% | 81.9558% | 82.6068% | 82.8632% |
| 2012 | 75.0555% | 82.2988% | 82.8808% | 83.2395% |
| 2013 | 79.353% | 82.6531% | 83.263% | 83.5776% |
| 2014 | 79.8199% | 83.4517% | 83.6635% | 83.9149% |
| 2015 | 80.9633% | 83.8602% | 84.1606% | 84.256% |
| 2016 | 81.6967% | 84.0668% | 84.3557% | 84.5153% |
| 2017 | 81.4923% | 84.2381% | 84.5327% | 84.6742% |
| 2018 | 81.9896% | 84.3966% | 84.6642% | 84.827% |
| 2019 | 82.0701% | 84.4683% | 84.7845% | 84.9741% |
| 2020 | 81.4659% | 84.5529% | 84.9057% | 85.0805% |
| 2021 | 81.788% | 84.6025% | 84.9481% | 85.1728% |
| 2022 | 81.5893% | 84.6307% | 84.9665% | 85.1863% |

Fig. S1. Overall accuracies achieved after applying the spatiotemporal filters on the initial (unfiltered) land-cover maps.

Table S2. Translation of classes from the Geo-spatial Application (GSA) to our classes.

| **Class code** | **Class name** | **GSA class and code** |
| --- | --- | --- |
| 110 | Winter cereals | Winter durum wheat (112), winter common wheat (115), spelt (120, 114), winter rye (121), winter barley (125,131), winter triticale (156) |
| 120 | Summer cereals | Summer barley (132), summer oats (143) |
| 130 | Maize | Grain maize (171), maize for biogas (172), silage maize (411) |
| 200 | Grassland | Meadows (451), mowing pastures (452), pastures (453), all other grassland uses (459), grassland reseeding (441), pasture reseeding (443), alpine pastures and meadows (455), vetches (221, 291), mixed legumes (250), forage grass (424), alfalfa (423), clovers (421, 422, 425, 426, 427), alfalfa-grass (433) |
| 1401 | Potato | Various potatoes (601, 602, 606, 614, 640, 641, 642, 643) |
| 1402 | Sugar beet | Sugar beet (603) |
| 1501 | Rapeseed | Winter rapeseed (311) |
| 1502 | Sunflower | Sunflowers (320) |
| 1601 | Legumes | Peas (210, 211, 212, 240), Broad bean (220), Lupin (230), Soybeans (330), lentils (292) |
| 1603 | Horticultural crops | Strawberries (707), asparagus (860), onion (633), pumpkin (630), carrot (634), garden bean (635), vegetables (610), cabbage (613), chicory (621), lettuce (637), spinach (638), other vegetables (632), carrot (183), cabbage (414), field vegetables (710), cauliflower (712), strawberries (723) |
| 3003 | Fallow land | Fallow lands (62, 66, 65, 560, 581, 582, 593, 594, 595, 590, 599) |
| 4001 | Vineyards | Vineyards (842, 843, 844, 847, 850, 851) |
| 4002 | Hops | Hops (750,856) |
| 4003 | Plantation | Pome and stone fruits, and fruit orchards (811, 814, 815, 816, 819, 824, 825, 826, 821, 829) |

Table S3. The accuracy of each agricultural mask from 1990 to 2022.

| **Year** | **Mask accuracy** |
| --- | --- |
| 1990 | 92.15% |
| 1991 | 92.27% |
| 1992 | 92.38% |
| 1993 | 92.44% |
| 1994 | 92.44% |
| 1995 | 92.43% |
| 1996 | 92.49% |
| 1997 | 92.52% |
| 1998 | 92.53% |
| 1999 | 92.51% |
| 2000 | 92.50% |
| 2001 | 92.49% |
| 2002 | 92.51% |
| 2003 | 92.58% |
| 2004 | 92.64% |
| 2005 | 92.70% |
| 2006 | 92.71% |
| 2007 | 92.77% |
| 2008 | 92.81% |
| 2009 | 92.78% |
| 2010 | 92.80% |
| 2011 | 92.83% |
| 2012 | 92.89% |
| 2013 | 92.97% |
| 2014 | 93.04% |
| 2015 | 93.13% |
| 2016 | 93.18% |
| 2017 | 93.18% |
| 2018 | 93.19% |
| 2019 | 93.20% |
| 2020 | 93.22% |
| 2021 | 93.19% |
| 2022 | 93.15% |

Fig. S2. Confusion matrix for 2010. The values in this and all subsequent confusion matrices are areas (km^2^). In all confusion matrices, OA = overall accuracy, UA = user’s accuracy, and PA = producer’s accuracy.

Fig. S3. Confusion matrix for 2011.

Fig. S4. Confusion matrix for 2012.

Fig. S5. Confusion matrix for 2013.

Fig. S6. Confusion matrix for 2014.

Fig. S7. Confusion matrix for 2015.

Fig. S8. Confusion matrix for 2016.

Fig. S9. Confusion matrix for 2017.

Fig. S10. Confusion matrix for 2018.

Fig. S11. Confusion matrix for 2019.

Fig. S12. Confusion matrix for 2020.

Fig. S13. Confusion matrix for 2021.

Fig. S14. Confusion matrix for 2022.

Fig. S15. Comparison of the F1-scores between our maps and other existing studies in 2016. In this figure and all F1-score figures hereafter, where the bar is empty for a particular study per class, it means that the class was not mapped or the data was not available for this year.

Fig. S16. Comparison of the F1-scores between our maps and other existing studies in 2017.

Fig. S17. Comparison of the F1-scores between our maps and other existing studies in 2018.

Fig. S18. Comparison of the F1-scores between our maps and other existing studies in 2019.

Fig. S19. Comparison of the F1-scores between our maps and other existing studies in 2020.

Fig. S20. Comparison of the F1-scores between our maps and other existing studies in 2021.

Fig. S21. Comparison of the F1-scores between our maps and other existing studies in 2022.

**Pseudocode 1. Model training**

Input:

- Multi-year training datasets
- Validation datasets
- Reference label classes

Output:

- Trained classification models
- Validation performance metrics

Workflow:

1. Define preprocessing functions
   a. Define function to normalize input observations to a common numerical range.
   b. Define the random observations selection (ROS) function to simulate missing observations.
   c. Define the random day shifting (RDS) function to simulate acquisition date variability.
2. Load training and validation datasets
   a. Read datasets corresponding to the selected years.
   b. Merge datasets when multiple years are used.
   c. Prepare feature arrays and class labels.
3. Initialize classification model
   a. Create the 1D-CNN model for temporal sequence classification.
   b. Configure optimizer and accuracy metric.
4. Repeat for each training epoch
   a. Shuffle training samples.
   b. Divide training data into mini-batches.
   c. For each training batch:
      i. Apply data augmentation in this order: ROS followed by RDS.
      ii. Normalize input features.
      iii. Convert labels to categorical representation.
      iv. Compute predictions.
      v. Calculate classification loss.
      vi. Update model parameters using gradient-based optimization.
   d. Evaluate model performance on validation data.
      i. Normalize validation features.
      ii. Generate predictions.
      iii. Compute validation loss and accuracy.
   e. Adapt optimization settings if validation performance stagnates.
   f. Store validation metrics and save the current model state.
5. Repeat until all epochs are completed.
6. Export trained models and performance statistics.

**Pseudocode 2. Model inference**

Input:

- Multi-temporal remote sensing observations
- Trained classification model
- Target year(s)
- Spatial processing units (tiles)

Output:

- Annual classification maps

Worflow

1. Select the trained model to be used for prediction.
   a. Retrieve model performance statistics.
   b. Identify the best-performing model according to validation metrics.
2. For each target year:
   a. Identify all spatial processing units to be classified.
3. For each spatial processing unit:
   a. Load all available observations for the target year.
   b. Extract the required feature layers.
   c. Organize observations into a temporally encoded annual sequence.
4. Apply preprocessing
   a. Convert temporal observations into the model input format.
   b. Normalize input features using the same procedure employed during training.
5. Perform model inference
   a. Process the data in manageable subsets.
   b. Generate class predictions for each pixel.
6. Post-process predictions
   a. Convert internal model classes to the target classification scheme.
7. Export outputs
   a. Save the annual classification map.
   b. Preserve spatial reference information.
8. Repeat for all spatial units and target years.
9. Mosaic outputs into the final map collection.

**Code 1. The 1D-CNN model created in Python using TensorFlow.**

**Parameter File 1. The preprocessing parameter file used to generate the ARD images using FORCE.**

++PARAM_TSA_START++

\# INPUT/OUTPUT DIRECTORIES

\# ------------------------------------------------------------------------

\# Lower Level datapool (parent directory of tiled input data)

\# Type: full directory path

DIR_LOWER = ~/input/BRDcube/Level2

\# Higher Level datapool (parent directory of tiled output data)

\# Type: full directory path

DIR_HIGHER = ~/ctmhistoric/TSS/

\# This is the directory where provenance files should be saved.

\# Type: full directory path

DIR_PROVENANCE = ~/ctmhistoric/TSS/prov/

\# MASKING

\# ------------------------------------------------------------------------

\# Analysis Mask datapool (parent directory of tiled analysis masks)

\# If no analsys mask should be applied, give NULL.

\# Type: full directory path

DIR_MASK = NULL

\# Basename of analysis masks (e.g. WATER-MASK.tif).

\# Masks need to be binary with 0 = off / 1 = on

\# Type: Basename of file

BASE_MASK = NULL

\# OUTPUT OPTIONS

\# ------------------------------------------------------------------------

\# Output format, which is either uncompressed flat binary image format aka

\# ENVI Standard, GeoTiff, or COG. GeoTiff images are compressed with LZW and hori-

\# zontal differencing; BigTiff support is enabled; the Tiff is structured

\# with striped blocks according to the TILE_SIZE (X) and BLOCK_SIZE (Y) speci-

\# fications. Metadata are written to the ENVI header or directly into the Tiff

\# to the FORCE domain. If the size of the metadata exceeds the Tiff's limit,

\# an external .aux.xml file is additionally generated.

\# Type: Character. Valid values: {ENVI,GTiff,COG,CUSTOM}

OUTPUT_FORMAT = GTiff

\# File that contains custom GDAL output options. This is only used if

\# OUTPUT_FORMAT = CUSTOM. If OUTPUT_FORMAT = CUSTOM, this file is mandatory.

\# The file should be written in tag and value notation. The first two lines

\# are mandatory and specify GDAL driver and file extension, e.g. DRIVER = GTiff

\# and EXTENSION = tif. The driver name refers to the GDAL short driver names.

\# Lines 3ff can hold a variable number of GDAL options (up to 32 are allowed).

\# Please note: with opening output options up to the user, it is now possible to

\# give invalid or conflicting options that result in the failure of creating files.

\# Type: full file path

FILE_OUTPUT_OPTIONS = NULL

\# This parameter controls whether the output is written as multi-band image, or

\# if the stack will be exploded into single-band files.

\# Type: Logical. Valid values: {TRUE,FALSE}

OUTPUT_EXPLODE = FALSE

\# PARALLEL PROCESSING

\# ------------------------------------------------------------------------

\# This module is using a streaming mechanism to speed up processing. There

\# are three processing teams (3 Threads) that simultaneously handle Input,

\# Processing, and Output. Example: when Block 2 is being processed, data

\# from Block 3 are already being input and results from Block 1 are being

\# output. Each team can have multiple sub-threads to speed up the work. The

\# number of threads to use for each team is given by following parameters.

\# Type: Integer. Valid range: [1,...

NTHREAD_READ = 30

NTHREAD_COMPUTE = 10

NTHREAD_WRITE = 10

\# PROCESSING EXTENT AND RESOLUTION

\# ------------------------------------------------------------------------

\# Analysis extent, given in tile numbers (see tile naming)

\# Each existing tile falling into this square extent will be processed

\# A shapefile of the tiles can be generated with force-tabulate-grid

\# Type: Integer list. Valid range: [-999,9999]

X_TILE_RANGE = 52 73

Y_TILE_RANGE = 38 63

\# Allow-list of tiles. Can be used to further limit the analysis extent to

\# non-square extents. The allow-list is intersected with the analysis extent,

\# i.e. only tiles included in both the analysis extent AND the allow-list will

\# be processed.

\# Optional. If NULL, the complete analysis extent is processed

\# Type: full file path

FILE_TILE = ~/tiles/ctmhist_tiles_de.til

\# This parameter can be used to override the default blocksize of the input

\# images (as specified in the datacube-definition.prj file). This can be

\# necessary if the default blocksize is too large for your system and you

\# cannot fit all necessary data into RAM. Note that processing of larger

\# blocksizes is more efficient. The tilesize must be dividable by the blocksize

\# without remainder. Set to 0, to use the default blocksize

\# Type: Double. Valid range: 0 or [RESOLUTION,TILE_SIZE]

BLOCK_SIZE = 0

\# Analysis resolution. The tile (and block) size must be dividable by this

\# resolution without remainder, e.g. 30m resolution with 100km tiles is not possible

\# Type: Double. Valid range: ]0,BLOCK_SIZE]

RESOLUTION = 30

\# How to reduce spatial resolution for cases when the image resolution is higher

\# than the analysis resolution. If FALSE, the resolution is degraded using Nearest

\# Neighbor resampling (fast). If TRUE, an approx. Point Spread Function (Gaussian

\# lowpass with FWHM = analysis resolution) is used to approximate the acquisition

\# of data at lower spatial resolution

\# Type: Logical. Valid values: {TRUE,FALSE}

REDUCE_PSF = TRUE

\# If you have spatially enhanced some Level 2 ARD using the FORCE Level 2 ImproPhe

\# module, this switch specifies whether the data are used at original (FALSE) or

\# enhanced spatial resolution (TRUE). If there are no improphe'd products, this

\# switch doesn't have any effect

\# Type: Logical. Valid values: {TRUE,FALSE}

USE_L2_IMPROPHE = FALSE

\# SENSOR ALLOW-LIST

\# ------------------------------------------------------------------------

\# Sensors to be used in the analysis. Multi-sensor analyses are restricted

\# to the overlapping bands. Following sensors are available: LND04 (6-band

\# Landsat 4 TM), LND05 (6-band Landsat 5 TM), LND07 (6-band Landsat 7 ETM+),

\# LND08/09 (6-band Landsat 8-9 OLI), SEN2A (10-band Sentinel-2A), SEN2B (10-band

\# Sentinel-2B), sen2a (4-band Sentinel-2A), sen2b (4-band Sentinel-2B),

\# S1AIA (2-band VV-VH Sentinel-1A IW ascending), S1BIA (2-band VV-VH Senti-

\# nel-1B IW ascending), S1AID (2-band VV-VH Sentinel-1A IW descending), S1BID

\# (2-band VV-VH Sentinel-1B IW descending), MOD01 (7-band Terra MODIS), MOD02.

\# (7-band Aqua MODIS).

\# The resulting outputs are named according to their band designation, i.e.

\# LNDLG ((6-band Landsat legacy bands), SEN2L (10-band Sentinel-2 land surface

\# bands), SEN2H (4-band Sentinel-2 high-res bands), R-G-B (3-band visual) or

\# VVVHP (VV/VH polarized), MODIS (7-band MODIS).

\# BAP Composites with such a band designation can be input again (e.g.

\# SENSORS = LNDLG).

\# Type: Character list. Valid values: {LND04,LND05,LND07,LND08,LND09,SEN2A,

\#	SEN2B,sen2a,sen2b,S1AIA,S1BIA,S1AID,S1BID,MOD01,MOD02,LNDLG,SEN2L,SEN2H,R-G-B,VVVHP,MODIS}

SENSORS = LND05 LND07 LND08 LND09 SEN2A SEN2B

\# Perform a spectral adjustment to Sentinel-2?

\# This method can only be used with following sensors: SEN2A, SEN2B, LND04, LND05, LND07,

\# LND08, LND09, MOD01, MOD02.

\# A material-specific spectral harmonization will be performed, which will convert the

\# spectral response of any of these sensors to Sentinel-2A. Non-existent bands will be

\# predicted, too.

\# Type: Logical. Valid values: {TRUE,FALSE}

SPECTRAL_ADJUST = FALSE

\# QAI SCREENING

\# ------------------------------------------------------------------------

\# This list controls, which QAI flags are masked out before doing the analysis.

\# Type: Character list. Valid values: {NODATA,CLOUD_OPAQUE,CLOUD_BUFFER,

\#	CLOUD_CIRRUS,CLOUD_SHADOW,SNOW,WATER,AOD_FILL,AOD_HIGH,AOD_INT,SUBZERO,

\#	SATURATION,SUN_LOW,ILLUMIN_NONE,ILLUMIN_POOR,ILLUMIN_LOW,SLOPED,WVP_NONE}

SCREEN_QAI = NODATA CLOUD_OPAQUE CLOUD_BUFFER CLOUD_CIRRUS CLOUD_SHADOW SNOW SUBZERO SATURATION

\# Threshold for removing outliers. Triplets of observations are used to determine

\# the overall noise in the time series by computinglinearly interpolating between

\# the bracketing observations. The RMSE of the residual between the middle value

\# and the interpolation is the overall noise. Any observations, which have a

\# residual larger than a multiple of the noise are iteratively filtered out

\# (ABOVE_NOISE). Lower/Higher values filter more aggressively/conservatively.

\# Likewise, any masked out observation (as determined by the SCREEN_QAI filter)

\# can be restored if its residual is lower than a multiple of the noise

\# (BELOW_NOISE). Higher/Lower values will restore observations more aggres-

\# sively/conservative. Give 0 to both parameters to disable the filtering.

\# Type: Float. Valid range: [0,...

ABOVE_NOISE = 0

BELOW_NOISE = 0

\# PROCESSING TIMEFRAME

\# ------------------------------------------------------------------------

\# Time extent for the analysis. All data between these dates will be used in

\# the analysis.

\# Type: Date list. Format: YYYY-MM-DD

DATE_RANGE = 1985-01-01 2023-12-31

\# DOY range for filtering the time extent. Day-of-Years that are outside of

\# the given interval will be ignored. Example: DATE_RANGE = 2010-01-01

\# 2019-12-31, DOY_RANGE = 91 273 will use all April-Sepember observations from

\# 2010-2019. If you want to extend this window over years give DOY min >

\# DOY max. Example: DATE_RANGE = 2010-01-01 2019-12-31, DOY_RANGE = 274 90

\# will use all October-March observations from 2010-2019.

\# Type: Integer list. Valid values: [1,365]

DOY_RANGE = 1 365

\# SPECTRAL INDEX

\# ------------------------------------------------------------------------

\# Perform the time series analysis using the specified band or index.

\# Multiple indices can be processed ar once to avoid multiple reads of the

\# same file. Only necessary bands will be input. You will be alerted if the

\# index cannot be computed based on the requested SENSORS. The index SMA is

\# a linear spectral mixture analysis and is dependent on the parameters

\# specified in the SPECTRAL MIXTURE ANALYSIS section below.

\# Type: Character list. Valid values: {BLUE,GREEN,RED,NIR,SWIR1,SWIR2,RE1,

\#	RE2,RE3,BNIR,NDVI,EVI,NBR,NDTI,ARVI,SAVI,SARVI,TC-BRIGHT,TC-GREEN,TC-WET,

\#	TC-DI,NDBI,NDWI,MNDWI,NDMI,NDSI,SMA,kNDVI,NDRE1,NDRE2,CIre,NDVIre1,NDVIre2,

\#	NDVIre3,NDVIre1n,NDVIre2n,NDVIre3n,MSRre,MSRren,CCI}

INDEX = NDVI NDWI SAVI BLUE GREEN RED NIR SWIR1 SWIR2

\# Standardize the TSS time series with pixel mean and/or standard deviation?

\# Type: Logical. Valid values: {NONE,NORMALIZE,CENTER}

STANDARDIZE_TSS = NONE

\# Output the quality-screened Time Series Stack? This is a layer stack of

\# index values for each date.

\# Type: Logical. Valid values: {TRUE,FALSE}

OUTPUT_TSS = TRUE

\# SPECTRAL MIXTURE ANALYSIS

\# ------------------------------------------------------------------------

\# This block only applies if INDEX includes SMA

\# ------------------------------------------------------------------------

\# Endmember file holding the endmembers according to the SENSORS band subset

\# Type: full file path

FILE_ENDMEM = NULL

\# Sum-to-One constrained unmixing?

\# Type: Logical. Valid values: {TRUE,FALSE}

SMA_SUM_TO_ONE = TRUE

\# Non-negativity constrained unmixing?

\# Type: Logical. Valid values: {TRUE,FALSE}

SMA_NON_NEG = TRUE

\# Apply shade normalization? If TRUE, the last endmember FILE_ENDMEM needs

\# to be the shade spectrum

\# Type: Logical. Valid values: {TRUE,FALSE}

SMA_SHD_NORM = TRUE

\# Endmember to be used for the analysis. This number refers to the column,

\# in which the desired endmember is stored (FILE_ENDMEM).

\# Type: Integer. Valid range: [1,NUMBER_OF_ENDMEMBERS]

SMA_ENDMEMBER = 1

\# Output the SMA model Error? This is a layer stack of model RMSE for

\# each date.

\# Type: Logical. Valid values: {TRUE,FALSE}

OUTPUT_RMS = FALSE

\# INTERPOLATION PARAMETERS

\# ------------------------------------------------------------------------

\# Interpolation method. You can choose between no, linear, moving average,

\# Radial Basis Function or harmonic Interpolation.

\# Harmonic interpolation can be used as a simple near-real time monitoring component.

\# Type: Character. Valid values: {NONE,LINEAR,MOVING,RBF,HARMONIC}

INTERPOLATE = NONE

\# Max temporal distance for the moving average filter in days. For each

\# interpolation date, MOVING_MAX days before and after are considered.

\# Type: Integer. Valid range: [1,365]

MOVING_MAX = 16

\# Sigma (width of the Gaussian bell) for the RBF filter in days. For each

\# interpolation date, a Gaussian kernel is used to smooth the observations.

\# The smoothing effect is stronger with larger kernels and the chance of

\# having nodata values is lower. Smaller kernels will follow the time series

\# more closely but the chance of having nodata values is larger. Multiple

\# kernels can be combined to take advantage of both small and large kernel

\# sizes. The kernels are weighted according to the data density within each

\# kernel.

\# Type: Integer list. Valid range: [1,365]

RBF_SIGMA = 8 16 32

\# Cutoff density for the RBF filter. The Gaussian kernels have infinite width,

\# which is computationally slow, and doesn't make much sense as observations

\# that are way too distant (in terms of time) are considered. Thus, the

\# tails of the kernel are cut off. This parameter specifies, which percen-

\# tage of the area under the Gaussian should be used.

\# Type: Float. Valid range: ]0,1]

RBF_CUTOFF = 0.95

\# Definition of how many modes per season are used for harmonic interpolation,

\# i.e. uni-modal (1), bi-modal (2), or tri-modal (3).

\# Type: Integer. Valid range: {1,2,3}

HARMONIC_MODES = 3

\# Subset of the time period to which the harmonic should be fitted.

\# For example, if the analysis timeframe is DATE_RANGE = 2015-01-01 2022-06-20,

\# all data from 2015-2022 will be considered. If HARMONIC_FIT_RANGE = 2015-01-01 2017-12-31,

\# the harmonic will only be fitted to the first 3 years of data.

\# Type: Date list. Format: YYYY-MM-DD

HARMONIC_FIT_RANGE = 2015-01-01 2017-12-31

\# Output of the near-real time product?

\# The product will contain the residual between the extrapolated harmonic and the actual data

\# following the defined end of the harmonic fit range.

\# This option requires harmonic interpolation (INTERPOLATE) and a forecast period (HARMONIC_FIT_RANGE).

\# Type: Logical. Valid values: {TRUE,FALSE}

OUTPUT_NRT = FALSE

\# This parameter gives the interpolation step in days.

\# Type: Integer. Valid range: [1,...

INT_DAY = 16

\# Standardize the TSI time series with pixel mean and/or standard deviation?

\# Type: Logical. Valid values: {NONE,NORMALIZE,CENTER}

STANDARDIZE_TSI = NONE

\# Output the Time Series Interpolation? This is a layer stack of index

\# values for each interpolated date. Note that interpolation will be per-

\# formed even if OUTPUT_TSI = FALSE - unless you specify INTERPOLATE = NONE.

\# Type: Logical. Valid values: {TRUE,FALSE}

OUTPUT_TSI = FALSE

\# PYTHON UDF PARAMETERS

\# ------------------------------------------------------------------------

\# This file specifies the file holding user-defined python code. You can skip this

\# by setting FILE_PYTHON = NULL, but this requires OUTPUT_PYP = FALSE.

\# Two functions are required to communicate with FORCE:

\# 0) The global space can be used to import modules etc.

\# 1) An initialization function that defines the number and names of output bands:

\#	``def forcepy_init(dates, sensors, bandnames):``

\# 2) A function that implements the user-defined functionality, see ``PYTHON_TYPE``

\# Type: full file path

FILE_PYTHON = NULL

\# Type of user-defined function.

\# 1) ``PIXEL`` expects a pixel-function that receives the time series of a single pixel

\# as 4D-nd.array [nDates, nBands, nrows, ncols]. A multi-processing pool is spawned to

\# parallely execute this function with ``NTHREAD_COMPUTE`` workers.

\#	``def forcepy_pixel(inarray, outarray, dates, sensors, bandnames, nodata, nproc):``

\# 2) ``BLOCK`` expects a pixel-function that receives the time series of a complete

\# processing unit as 4D-nd.array [nDates, nBands, nrows, ncols]. No parallelization is

\# done on FORCE's end.

\#	``def forcepy_block(inblock, outblock, dates, sensors, bandnames, nodata, nproc):``

\# Type: Character. Valid values: {PIXEL,BLOCK}

PYTHON_TYPE = PIXEL

\# Output the results provided by the python UDF? If TRUE, FILE_PYTHON must exist.

\# Type: Logical. Valid values: {TRUE,FALSE}

OUTPUT_PYP = FALSE

\# SPECTRAL TEMPORAL METRICS

\# ------------------------------------------------------------------------

\# Output Spectral Temporal Metrics? The remaining parameters in this block

\# are only evaluated if TRUE

\# Type: Logical. Valid values: {TRUE,FALSE}

OUTPUT_STM = FALSE

\# Which Spectral Temporal Metrics should be computed? The STM output files

\# will have as many bands as you specify metrics (in the same order).

\# Currently available statistics are the average, standard deviation, mini-

\# mum, maximum, range, skewness, kurtosis, any quantile from 1-99%, and

\# interquartile range. Note that median is Q50.

\# Type: Character list. Valid values: {MIN,Q01-Q99,MAX,AVG,STD,RNG,IQR,SKW,KRT,NUM}

STM = Q25 Q50 Q75 AVG STD

\# FOLDING PARAMETERS

\# ------------------------------------------------------------------------

\# Which statistic should be used for folding the time series? This parameter

\# is only evaluated if one of the following outputs in this block is requested.

\# Currently available statistics are the average, standard deviation, mini-

\# mum, maximum, range, skewness, kurtosis, median, 10/25/75/90% quantiles,

\# and interquartile range

\# Type: Character. Valid values: {MIN,Q10,Q25,Q50,Q75,Q90,MAX,AVG,STD,

\#	RNG,IQR,SKW,KRT,NUM

FOLD_TYPE = AVG

\# Standardize the FB* time series with pixel mean and/or standard deviation?

\# Type: Logical. Valid values: {NONE,NORMALIZE,CENTER}

STANDARDIZE_FOLD = NONE

\# Output the Fold-by-Year/Quarter/Month/Week/DOY time series? These are layer

\# stacks of folded index values for each year, quarter, month, week or DOY.

\# Type: Logical. Valid values: {TRUE,FALSE}

OUTPUT_FBY = FALSE

OUTPUT_FBQ = FALSE

OUTPUT_FBM = FALSE

OUTPUT_FBW = FALSE

OUTPUT_FBD = FALSE

\# Compute and output a linear trend analysis on any of the folded time series?

\# Note that the OUTPUT_FBX parameters don't need to be TRUE to do this.

\# See also the TREND PARAMETERS block below.

\# Type: Logical. Valid values: {TRUE,FALSE}

OUTPUT_TRY = FALSE

OUTPUT_TRQ = FALSE

OUTPUT_TRM = FALSE

OUTPUT_TRW = FALSE

OUTPUT_TRD = FALSE

\# Compute and output an extended Change, Aftereffect, Trend (CAT) analysis on

\# any of the folded time series?

\# Note that the OUTPUT_FBX parameters don't need to be TRUE to do this.

\# See also the TREND PARAMETERS block below.

\# Type: Logical. Valid values: {TRUE,FALSE}

OUTPUT_CAY = FALSE

OUTPUT_CAQ = FALSE

OUTPUT_CAM = FALSE

OUTPUT_CAW = FALSE

OUTPUT_CAD = FALSE

\# LAND SURFACE PHENOLOGY PARAMETERS - SPLITS-BASED

\# ------------------------------------------------------------------------

\# The Land Surface Phenology (LSP) options are only available if FORCE was

\# compiled with SPLITS (see installation section in the FORCE user guide).

\# ------------------------------------------------------------------------

\# For estimating LSP for one year, some data from the previous/next year

\# need to be considered to find the seasonal minima, which define a season.

\# The parameters are given in DOY, i.e. LSP_DOY_PREV_YEAR = 273, and

\# LSP_DOY_NEXT_YEAR = 91 will use all observations from October (Year-1)

\# to March (Year+1)

\# Type: Integer. Valid range: [1,365]

LSP_DOY_PREV_YEAR = 273

LSP_DOY_NEXT_YEAR = 91

\# Seasonality is of Northern-, Southern-hemispheric or of mixed type? If

\# mixed, the code will attempt to estimate the type on a per-pixel basis.

\# Type: Character. Valid values: {NORTH,SOUTH,MIXED}

LSP_HEMISPHERE = NORTH

\# How many segments per year should be used for the spline fitting? More

\# segments follow the seasonality more closely, less segments smooth the

\# time series stronger.

\# Type: Integer. Valid range: [1,...

LSP_N_SEGMENT = 4

\# Amplitude threshold for detecing Start, and End of Season, i.e. the date,

\# at which xx% of the amplitude is observed

\# Type: Float. Valid range: ]0,1[

LSP_AMP_THRESHOLD = 0.2

\# LSP won't be derived if the seasonal index values do not exceed following

\# value. This is useful to remove unvegetated surfaces.

\# Type: Integer. Valid range: [-10000,10000]

LSP_MIN_VALUE = 500

\# LSP won't be derived if the seasonal amplitude is below following value

\# This is useful to remove surfaces that do not have a seasonality.

\# Type: Integer. Valid range: [0,10000]

LSP_MIN_AMPLITUDE = 500

\# Which Phenometrics should be computed? There will be a LSP output file for

\# each metric (with years as bands).

\# Currently available are the dates of the early minimum, start of season,

\# rising inflection, peak of season, falling inflection, end of season, late

\# minimum; lengths of the total season, green season; values of the early

\# minimum, start of season, rising inflection, peak of season, falling

\# inflection, end of season, late minimum, base level, seasonal amplitude;

\# integrals of the total season, base level, base+total, green season; rates

\# of averahe rising, average falling, maximum rising, maximum falling.

\# Type: Character list. Valid values: {DEM,DSS,DRI,DPS,DFI,DES,DLM,LTS,LGS,

\#	VEM,VSS,VRI,VPS,VFI,VES,VLM,VBL,VSA,IST,IBL,IBT,IGS,RAR,RAF,RMR,RMF}

LSP = VSS VPS VES VSA RMR IGS

\# Standardize the LSP time series with pixel mean and/or standard deviation?

\# Type: Logical. Valid values: {NONE,NORMALIZE,CENTER}

STANDARDIZE_LSP = NONE

\# Output the Spline fit? This is a layer stack of fitted index values for

\# interpolated date.

\# Type: Logical. Valid values: {TRUE,FALSE}

OUTPUT_SPL = FALSE

\# Output the Phenometrics? These are layer stacks per phenometric with as many

\# bands as years (excluding one year at the beginning/end of the time series.

\# Type: Logical. Valid values: {TRUE,FALSE}

OUTPUT_LSP = FALSE

\# Compute and output a linear trend analysis on the requested Phenometric time

\# series? Note that the OUTPUT_LSP parameters don't need to be TRUE to do this.

\# See also the TREND PARAMETERS block below.

\# Type: Logical. Valid values: {TRUE,FALSE}

OUTPUT_TRP = FALSE

\# Compute and output an extended Change, Aftereffect, Trend (CAT) analysis on

\# the requested Phenometric time series?

\# Note that the OUTPUT_LSP parameters don't need to be TRUE to do this.

\# See also the TREND PARAMETERS block below.

\# Type: Logical. Valid values: {TRUE,FALSE}

OUTPUT_CAP = FALSE

\# LAND SURFACE PHENOLOGY PARAMETERS - POLAR-BASED

\# ------------------------------------------------------------------------

\# Please note that these only work well with non-negative indices.--------

\# ------------------------------------------------------------------------

\# Threshold for detecing Start of Season in the cumulative time series.

\# Type: Float. Valid range: ]0,1[

POL_START_THRESHOLD = 0.2

\# Threshold for detecing Mid of Season in the cumulative time series.

\# Type: Float. Valid range: ]0,1[

POL_MID_THRESHOLD = 0.5

\# Threshold for detecing End of Season in the cumulative time series.

\# Type: Float. Valid range: ]0,1[

POL_END_THRESHOLD = 0.8

\# Should the start of each phenological year be adapated?

\# If FALSE, the start is static, i.e. Date of Early Minimum and Date of Late

\# Minimum are the same for all years and 365 days apart. If TRUE, they differ

\# from year to year and a phenological year is not forced to be 365 days long.

\# Type: Logical. Valid values: {TRUE,FALSE}

POL_ADAPTIVE = TRUE

\# Which Polarmetrics should be computed? There will be a POL output file for

\# each metric (with years as bands).

\# Currently available are the dates of the early minimum, late minimum, peak of season,

\# start of season, mid of season, end of season, early average vector, average vector,

\# late average vector; lengths of the total season, green season, between averge vectors;

\# values of the early minimum, late minimum, peak of season, start of season, mid of season,

\# end of season, early average vector, average vector, late average vector, base level,

\# green amplitude, seasonal amplitude, peak amplitude, green season mean , green season

\# variability, dates of start of phenological year, difference between start of phenological

\# year and its longterm average; integrals of the total season, base level, base+total,

\# green season, rising rate, falling rate; rates of average rising, average falling, maximum

\# rising, maximum falling.

\# Type: Character list. Valid values: {DEM,DLM,DPS,DSS,DMS,DES,DEV,DAV,DLV,LTS,

\#	LGS,LGV,VEM,VLM,VPS,VSS,VMS,VES,VEV,VAV,VLV,VBL,VGA,VSA,VPA,VGM,VGV,DPY,DPV,

\#	IST,IBL,IBT,IGS,IRR,IFR,RAR,RAF,RMR,RMF}

POL = VSS VPS VES VSA RMR IGS

\# Standardize the POL time series with pixel mean and/or standard deviation?

\# Type: Logical. Valid values: {NONE,NORMALIZE,CENTER}

STANDARDIZE_POL = NONE

\# Output the polar-transformed time series? These are layer stack of cartesian X-

\# and Y-coordinates for each interpolated date. This results in two files, product

\# IDs are PCX and PCY.

\# Type: Logical. Valid values: {TRUE,FALSE}

OUTPUT_PCT = FALSE

\# Output the Polarmetrics? These are layer stacks per polarmetric with as many

\# bands as years.

\# Type: Logical. Valid values: {TRUE,FALSE}

OUTPUT_POL = FALSE

\# Compute and output a linear trend analysis on the requested Polarmetric time

\# series? Note that the OUTPUT_POL parameters don't need to be TRUE to do this.

\# See also the TREND PARAMETERS block below.

\# Type: Logical. Valid values: {TRUE,FALSE}

OUTPUT_TRO = FALSE

\# Compute and output an extended Change, Aftereffect, Trend (CAT) analysis on

\# the requested Polarmetric time series?

\# Note that the OUTPUT_POL parameters don't need to be TRUE to do this.

\# See also the TREND PARAMETERS block below.

\# Type: Logical. Valid values: {TRUE,FALSE}

OUTPUT_CAO = FALSE

\# TREND PARAMETERS

\# ------------------------------------------------------------------------

\# This parameter specifies the tail-type used for significance testing of

\# the slope in the trend analysis. A left-, two-, or right-tailed t-test

\# is performed.

\# Type: Character. Valid values: {LEFT,TWO,RIGHT}

TREND_TAIL = TWO

\# Confidence level for significance testing of the slope in the trend analysis

\# Type: Float. Valid range: [0,1]

TREND_CONF = 0.95

\# In the Change, Aftereffect, Trend (CAT) analysis: do you want to

\# put a penalty on non-permanent change for the change detection?

\# This can help to reduce the effect of outliers.

\# Type: Logical. Valid values: {TRUE,FALSE}

CHANGE_PENALTY = FALSE

++PARAM_TSA_END++
